# APUM5, encoding a Pumilio RNA binding protein, negatively regulates abiotic stress responsive gene expression

**DOI:** 10.1186/1471-2229-14-75

**Published:** 2014-03-25

**Authors:** Sung Un Huh, Kyung-Hee Paek

**Affiliations:** 1 College of Life Sciences and Biotechnology, Korea University, 1, 5-ga, Anam-dong, Sungbuk-gu, Seoul 136-701, Republic of Korea; 2 Present address: The Sainsbury Laboratory, Norwich Research Park, Norwich NR4 7UH, UK

## Abstract

**Background:**

A mutant screening was carried out previously to look for new genes related to the *Cucumber mosaic virus* infection response in *Arabidopsis*. A Pumilio RNA binding protein-coding gene, *Arabidopsis Pumilio RNA binding protein 5* (*APUM5*), was obtained from this screening.

**Results:**

*APUM5* transcriptional profiling was carried out using a bioinformatics tool. We found that *APUM5* was associated with both biotic and abiotic stress responses. However, bacterial and fungal pathogen infection susceptibility was not changed in *APUM5* transgenic plants compared to that in wild type plants although *APUM5* expression was induced upon pathogen infection. In contrast, *APUM5* was involved in the abiotic stress response. *35S*-*APUM5* transgenic plants showed hypersensitive phenotypes under salt and drought stresses during germination, primary root elongation at the seedling stage, and at the vegetative stage in soil. We also showed that some abiotic stress-responsive genes were negatively regulated in *35S*-*APUM5* transgenic plants. The APUM5-Pumilio homology domain (PHD) protein bound to the 3′ untranslated region (UTR) of the abiotic stress-responsive genes which contained putative Pumilio RNA binding motifs at the 3′ UTR.

**Conclusions:**

These results suggest that APUM5 may be a new post-transcriptional regulator of the abiotic stress response by direct binding of target genes 3′ UTRs.

## Background

Post-transcriptional/translational control of gene expression is a powerful strategy for the eukaryotes to adapt to biotic and abiotic stresses. In particular, this process is regulated by various RNA-binding proteins (RBPs) that regulate several processes involving RNA processing, mRNA transport, mRNA stability, and mRNA translation through direct or indirect interactions with target mRNAs [1-5]. Many RBPs have several conserved RNA binding domains (RBDs) to interact with target RNAs; these are the RNA-recognition motif (RRM), the K Homology domain, the Pumilio homology domain (PHD), and the double-stranded RNA binding domain [6-8].

The Pumilio RNA binding family proteins termed Puf proteins have repeats of a conserved PHD, which recognizes a highly conserved 8–10 nucleotide core motif including the ‘UGUA’ tetranucleotide in the 3′ mRNA untranslated region (UTR) to specifically regulate target mRNA stability and translation. Mutated key amino acids of this PHD alter the sequence specificity of Pumilio 1, a human Pumilio protein 1 [8,9]. Most of the identified Puf proteins are conserved throughout evolution in mammals, fungi, protozoa, and plants [10-13]. Many Puf proteins have been identified as essential factors for several aspects of development such as germline stem cell maintenance, synaptic plasticity, embryonic axis patterning, and mating type switching [13-17]. Many approaches such as DNA microarrays, bioinformatics approaches, and RNA immunoprecipitation have been performed in *Saccharomyces cerevisiae* and *Drosophila melanogaster* to identify specific target RNAs associated with Puf proteins [15,18,19]. Many RNAs with binding motifs interact with Puf proteins at different developmental stages and in different tissues. Puf proteins may be involved in much larger and richer post-transcriptional regulation in mammals. However, it is largely unknown how plant Pufs control post-transcriptional/translational processing by binding to their target 3′ UTR transcripts, and their functions have been poorly analyzed.

Recent work has identified putative mRNA targets of *Arabidopsis* Pumilio (APUM) proteins, and a comparative analysis of plant Puf proteins was performed [11,20]. These results demonstrate that plant Puf proteins may also act as post-transcriptional/translational repressors through an evolutionarily conserved mechanism, and their recombinant PHD protein binds to the nanos response element (NRE) sequence within the 3′ UTR of *hunchback* (*hb*) mRNA of the *Drosophila* Pumilio target. Additionally, their putative target RNA candidates are associated with plant growth and development similar to mammalian Puf proteins [11,20]. Some Puf proteins affect rRNA processing apparently without target sequence specificity. For example, analysis of *APUM23* knock-out plant phenotypes revealed that *APUM23* function is involved in rRNA processing in the *Arabidopsis* nucleolar region [21].

We previously isolated an *Arabidopsis Pumilio RNA binding protein 5* (*APUM5*) mutant as a susceptibility-reduced mutant to *Cucumber mosaic virus* (CMV) infection and this mutant exhibits up-regulated *APUM5* gene expression [22]. APUM5 has a conserved eight tandem repeats of PHD but their function in *Arabidopsis* is still largely unknown. Thus, we analyzed *APUM5* gene expression patterns using a bioinformatics tool and found it could be up-regulated by biotic and abiotic stresses. In this study, we conducted loss-of-function and gain-of-function studies using *APUM5* transgenic plants to further elucidate the specific roles and regulation of APUM5 under biotic and abiotic stress conditions. The results showed that APUM5 functions as a negative regulator under salt, osmotic, and drought stress conditions although *APUM5* transgenic plants did not show any phenotypic change upon bacterial and fungal pathogen infections.

## Results

### *APUM5* is a pathogen-responsive gene but does not affect susceptibility or resistance to *Pseudomonas syringae* pv*. tomato* DC3000 (*Pst* DC3000) and *Alternaria brassicicola* infections

**Figure 1:**
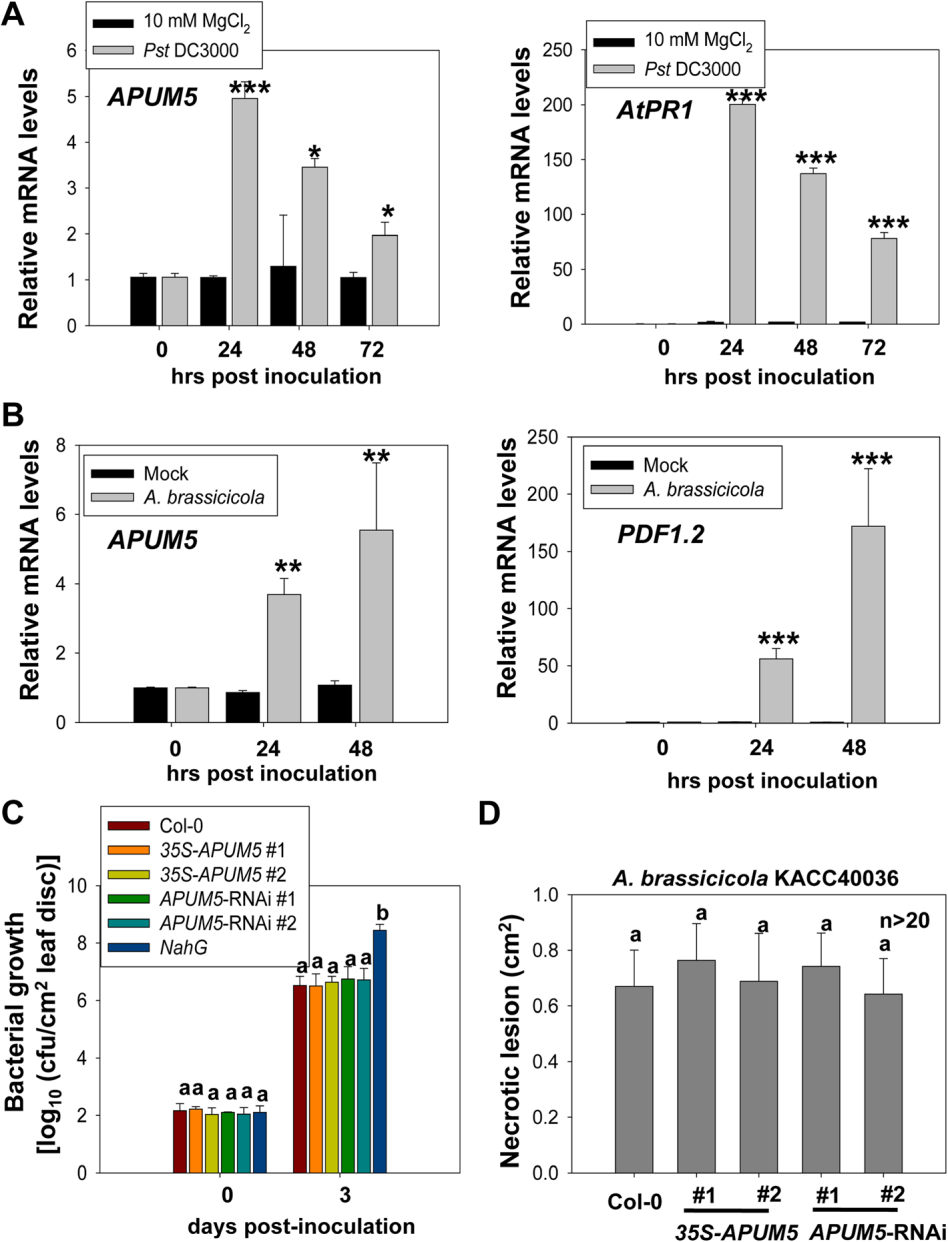
***APUM5 *was induced in response to *Pst *DC3000 and *A. brassicicola *infection but did not affect susceptibility. (A)***APUM5* gene expression pattern upon *Pst* DC3000 inoculation. *AtPR1* was used as a positive control for *Pst* DC3000 inoculation. Error bars indicate standard deviations (n = 3). (Student’s *t*-test; *P < 0.01, ***P < 0.0001). **(B)***APUM5* was induced by *A. brassicicola* infection. *PDF1.2* was used as a positive control for fungal pathogen infection. Error bars indicate standard deviations (n = 3). (Student’s *t*-test; **P < 0.001, ***P < 0.0001). **(C)** Bacterial growth analysis in *APUM5* transgenic plants. *NahG* plant was used as a positive control for *Pst* DC3000 inoculation. Error bars indicate standard deviations (n = 3). **(D)** Quantitative analysis of necrotic lesion size upon *A. brassicicola* infection at 5 dpi.

APUM5 is involved in susceptibility to CMV [22,23]. An *APUM5* gene transcript analysis was performed via the *Arabidopsis* eFP Browser to further elucidate the functional role of APUM5 [24]. Interestingly, *APUM5* was induced by biotic and abiotic stresses in the bioinformatics analysis. Furthermore, *APUM5* gene expression was enhanced in the *mpk4*-*1* mutant background compared with accession Landsberg *erecta* (L*er*) control plants (Additional file 1). *Arabidopsis* MAP kinase 4 (AtMPK4) is a negative regulator involved in salicylic acid-dependent disease resistance. *mpk4-1* exhibits enhanced resistance to biotrophs and increased susceptibility to necrotrophs [25]. Based on these results, we postulated that APUM5 might be associated with the biotic/abiotic defense response pathway. First, we confirmed whether *APUM5* expression is responsive to infection by bacterial pathogens such as *Pst* DC3000. As a result, *APUM5* mRNA expression increased upon *Pst* DC3000 infection at 24 h post infection (hpi) and started to diminish at 48 and 72 hpi (Figure 1A). *AtPR1* was used as a positive control and was successfully induced upon *Pst* DC3000 infection (Figure 1A). Thus, we checked the possibility that APUM5 might be involved in the pathogen defense response and could affect bacterial growth. However, *APUM5* transgenic plants (both *35S*-*APUM5* and *APUM5*-RNAi plants) did not show changes in susceptibility or resistance in the *Pst* DC3000 growth assay (Figure 1C). These data indicate that APUM5 is not required for bacterial pathogen resistance although *APUM5* was induced upon bacterial infection.

Next, we checked whether *APUM5* affected fungal pathogen infection. When Col-0 plants were inoculated with *A. brassicicola*, *APUM5* mRNA expression levels increased at the 24 and 48 h time points (Figure 1B). *PDF1.2*, which is a positive control for fungal pathogen infection, was induced by the *A. brassicicola* infection (Figure 1B). We evaluated the necrotic lesion size in Col-0 and *APUM5* transgenic plants upon *A. brassicicola* infection. The *APUM5* transgenic plants did not exhibit any significant increase or decrease in necrotic lesion sizes compared with those in Col-0 plants (Figure 1D). Thus, *APUM5* overexpression or knockdown did not change the fungal pathogen growth phenotype, although *APUM5* is a fungal pathogen-responsive gene. These results suggest that APUM5 is not associated with defense against *Pst* DC3000 and *A. brassicicola* infections even though *APUM5* expression response to bacterial and fungal pathogens.

### Expression of *APUM5* increases strongly following mannitol, salt, and ABA treatments

We found that *APUM5* is a pathogen-responsive gene following bacterial and fungal pathogen infections. However, the function of *APUM5* was not associated with defense against these pathogens, although APUM5 inhibits CMV infection by binding to the CMV 3′ UTR [22,23]. Furthermore, *APUM5* transgenic plants did not show enhanced or repressed *PR* gene expression [22]. These results might explain that APUM5 did not directly regulate defense-related genes.

**Figure 2:**
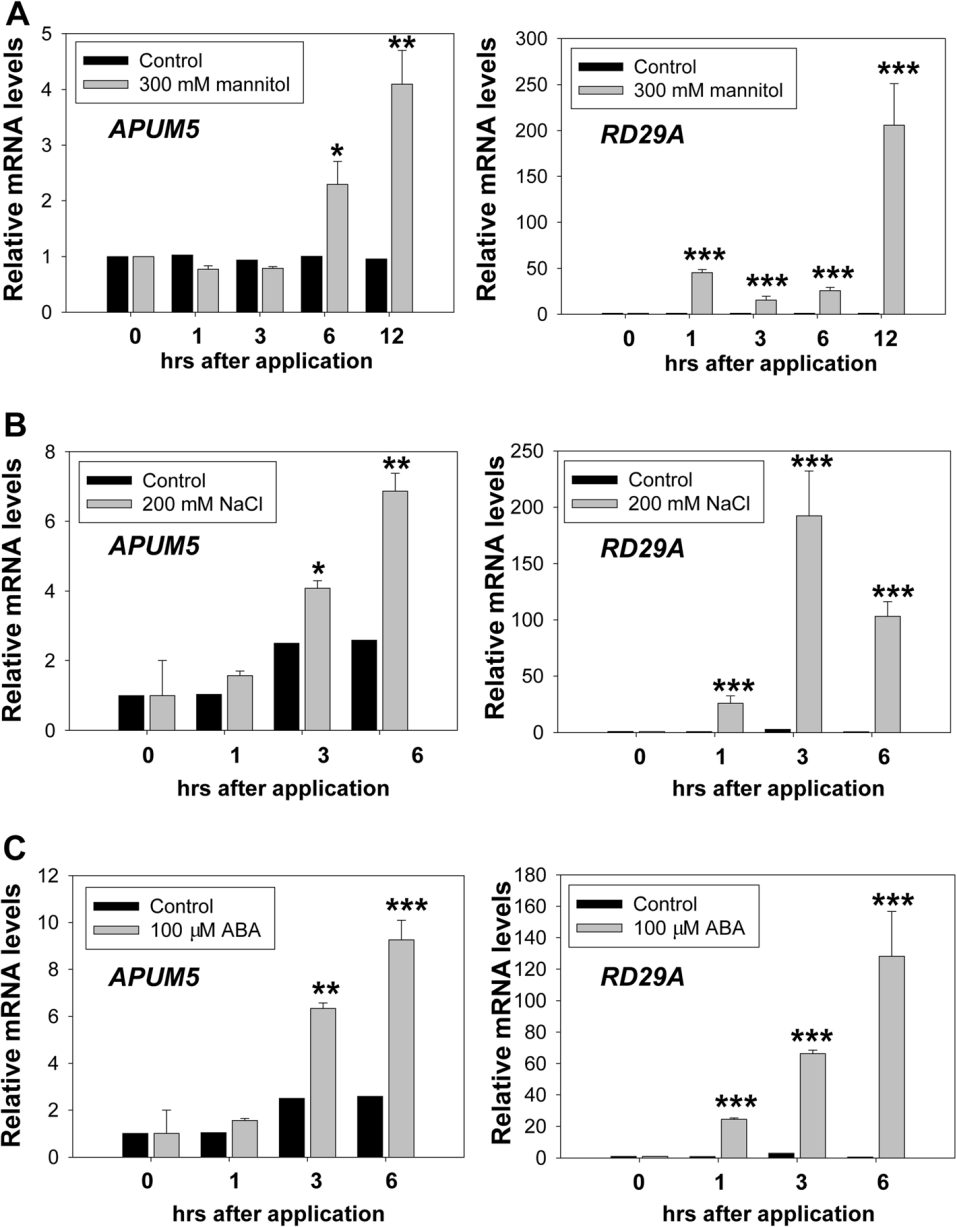
**Expression analysis of *APUM5 *by qRT-PCR following osmotic stress, salt, and ABA treatments. (A)** Expression patterns of *APUM5 and RD29A* in response to mannitol treatment. **(B)** Expression patterns of *APUM5* and *RD29A* in response to salt treatment. **(C)** Transcription levels of *APUM5* and *RD29A* following ABA treatment. Expression patterns of *RD29A* were used as a positive control for the mannitol, NaCl, and ABA treatments. *APUM5* and *RD29A* transcription levels in 10-day-old wild-type *Arabidopsis* seedlings grown on 1/2 MS medium containing 1.5% sucrose and treated without (control) or with 300 mM mannitol, 200 mM NaCl, or 100 μM ABA were analyzed by qRT-PCR. Error bars indicate standard deviations (n = 3). (Student’s *t*-test; *P < 0.01, **P < 0.001, ***P < 0.0001).

*APUM5* is significantly induced by abiotic stressors such as mannitol, salt, ABA treatments [24]. We verified that *APUM5* expression increased rapidly in response to mannitol treatment in 10-day-old seedlings (Figure 2A). The *RD29A* promoter contains both a dehydration-responsive element (*DRE*) and an ABA-responsive element (*ABRE*), which are two major *cis*-acting elements involved in ABA-independent and -dependent gene expression, respectively [26]. *RD29A* was used as a positive control and was successfully induced by mannitol stress (Figure 2A). To further investigate the effects of other osmotic stressors and ABA treatment on *APUM5* expression, the expression levels of *APUM5* in 10-day-old seedlings treated with NaCl and ABA were measured by quantitative reverse transcription-polymerase chain reaction (qRT-PCR). *APUM5* expression increased gradually and strongly following NaCl treatment (Figure 2B). We also evaluated the effect of ABA. As expected, *APUM5* gene expression was enhanced by ABA treatment (Figure 2C). *RD29A* expression was also increased by the NaCl and ABA treatments (Figure 2). These observations suggest that APUM5 may play a role during the osmotic and ABA stress response.

### Tissue-specific expression of GUS in *APUM5pro*-*GUS* transgenic plants

**Figure 3:**
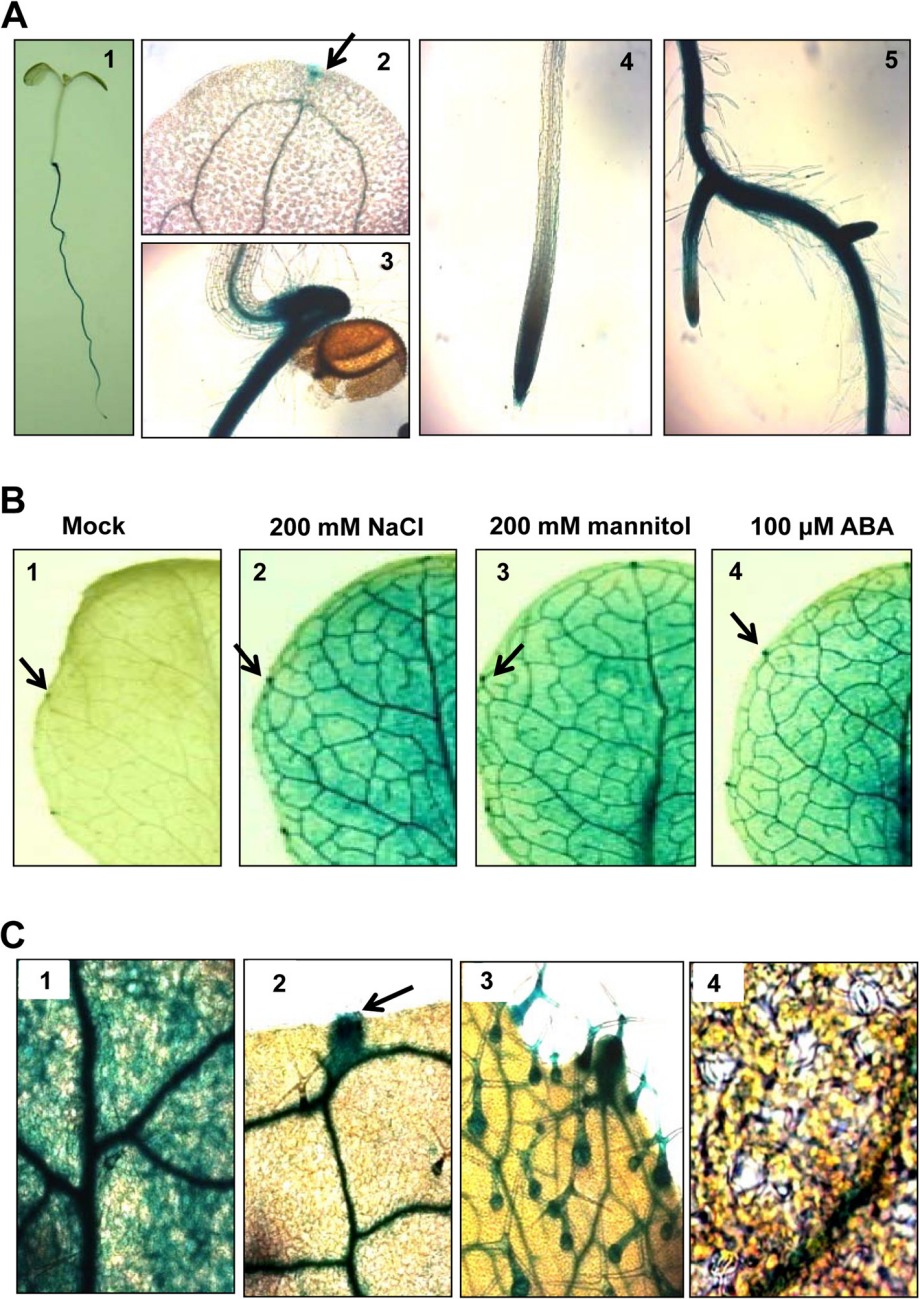
**Tissue-specific expression of GUS in *APUM5pro*-*GUS *transgenic plants. (A)***APUM5* promoter activity was determined by GUS histochemical staining. Section 1, 4-day-old seedlings. Section 2, hydathode on the cotyledon indicated by an arrow. Section 3, shoot apical meristem region. Section 4, primary root. Section 5, lateral roots. **(B)** The effect of salt, osmotic, and ABA treatments on *APUM5pro*-*GUS* activity. Three-week-old seedlings were treated with 200 mM NaCl, 200 mM mannitol, or 100 μM ABA for 6 h before GUS staining. Enhanced *APUM5* promoter activity on the rosette leaf following abiotic stress. **(C)** Enlarged image from 3B. Section 1, *APUM5* promoter activity expressed in leaf vasculature and mesophyll cell regions. Section 2, hydathode and leaf vascular bundle. Section 3, trichomes on a rosette leaf. Section 4, guard cells.

A 1.3-kb fragment of the *APUM5* promoter region was fused to the *GUS* reporter gene, and this construct was introduced into *Arabidopsis* to analyze the spatial expression of *APUM5. APUM5pro*-*GUS* expression was analyzed in the 10-day-old seedling stage of T3 transgenic plants, with strong GUS activity in the root tip, primary root, lateral root, and shoot apical meristem region (Figure 3A, Sections 1, 3, 4, and 5). Although low GUS activity was detected in leaf tissue, much higher activity was observed in the hydathodal cells (Figure 3A, Section 2). High levels of GUS activity were also detected in cauline leaves, flowers, and silique ends (Additional file 2). These observations confirmed results described previously [27].

Previous results showed that *APUM5* expression was highly enhanced under the osmotic stress condition (Figure 2), indicating that *APUM5* expression could be observed in the leaf tissue in the GUS activity assay following osmotic stress treatment. Thus, the *APUM5* promoter *cis*-elements were examined using Athena, the *Arabidopsis* promoter analysis tool [28]. The promoter analysis revealed that the *APUM5* promoter contains ABA response elements (ABFs binding motif, *ABREATRD22*) and MYB recognition elements (MYB4 binding motif, *MYB1AT*), all of which are *cis*-acting elements often found in ABA- or environmental stress-related genes (Additional file 3) [26]. *APUM5pro-GUS* transgenic plants were treated with mannitol, NaCl, and exogenous ABA in soil to further investigate *APUM5* expression. After applying of NaCl, mannitol, and ABA, most of the leaf tissues showed strong GUS activity and were activated in the entire plant vasculature (Figure 3B, C). This was consistent with qRT-PCR analyses of the *APUM5* gene expression pattern during osmotic stress and exogenous ABA applications (Figure 2). Interestingly, GUS activity was not detected in guard cells (Figure 3C, Section 4), whereas strong GUS activity was detected in the hydathodes, trichomes, mesophyll cells, main veins, and vascular tissues (Figure 3C, Sections 1–3). These results indicate that APUM5 may not directly affect stomatal regulation through an ABA-dependent pathway of stomatal closure and opening. In contrast, APUM5 may affect the physical endurance of the plants under osmotic or drought stress via hydathodes and trichomes.

### Overexpression of *APUM5* leads to hypersensitivity and down-regulation to reduced susceptibility to salt and mannitol in *Arabidopsis*

**Figure 4:**
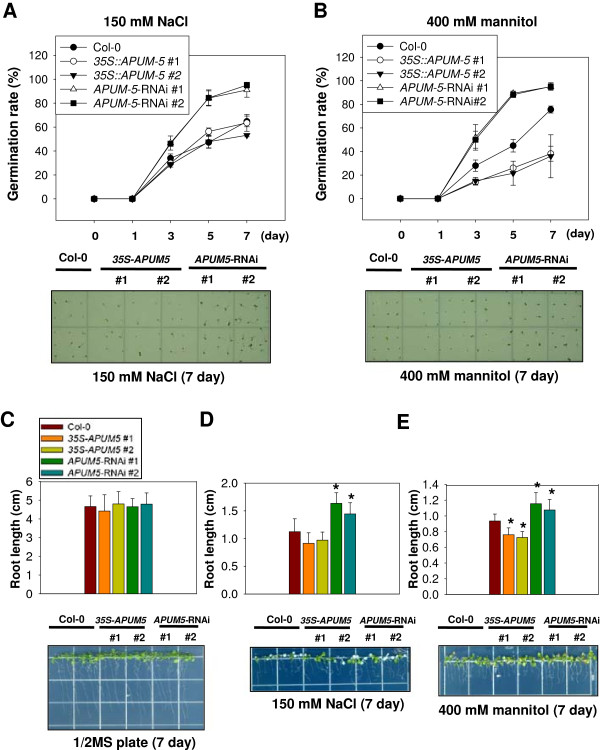
**Effect of salt and dehydration stress on growth of wild-type and *APUM5 *transgenic seedlings. (A)** The germination ability of wild-type, *35S*-*APUM5*, and *APUM5*-RNAi plants was measured on 1/2 MS medium containing 150 mM NaCl, and germination was scored at the indicated days. **(B)** The germination test of wild-type, *35S*-*APUM5*, and *APUM5*-RNAi transgenic plants upon 1/2 MS medium containing 400 mM mannitol. These experiments were performed for three independent sets (60–70 seeds per experiment). **(C)** Normal root growth of Col-0 and *APUM5* transgenic plants. **(D)** The effect of 150 mM NaCl on primary root elongation. **(E)** The effect of 400 mM mannitol on primary root elongation. Seeds were germinated for 3 days on 1/2 MS medium and the seedlings were transferred (n = 30, triplicates) to 1/2 MS containing 150 mM NaCl or 400 mM mannitol. Primary root length was measured 7 days after transfer. Error bars indicate standard deviations (n = 3). (Student’s *t*-test; *P < 0.01).

The above results demonstrated that osmotic stress and exogenous ABA treatment up-regulated *APUM5* expression (Figure 2) and this result was consistent with *APUM5pro*-*GUS* expression under similar stress conditions (Figure 3B and C). Additionally, these results are supported by the *APUM5* promoter *cis*-acting elements analysis (Additional file 3). These data suggest that APUM5 may be involved in osmotic, drought, and ABA sensitivity. Germination and post-germination growth efficiency of *APUM5* transgenic and Col-0 wild-type plants were examined when the plants were treated with various concentrations of salt or mannitol to determine whether the physiological role of APUM5 in *Arabidopsis* is associated with salt or dehydration stress. The germination rate of Col-0, *35S*-*APUM5*, and *APUM5*-RNAi plants was similar on the control 1/2 MS medium plate (Additional file 4). However, the germination rate of *APUM5*-RNAi line #1 and #2 plants was about 20% higher than that of Col-0 on the 1/2 MS medium supplemented with 150 mM NaCl, whereas no obvious difference in germination rate was observed between the Col-0 and *35S*-*APUM5* transgenic plants (Figure 4A). However, primary root elongation of *35S*-*APUM5* transgenic plants was hypersensitive in the 1/2 MS plate containing 100 mM NaCl (Additional file 5A). On the other hand, the germination rate of *APUM5*-RNAi line #1 and #2 plants increased about 18% compared with that of the Col-0 plants on 1/2 MS medium supplemented with 400 mM mannitol, whereas the germination rate of *35S-APUM5* transgenic line #1 and #2 plants decreased significantly (about 35%) by 7 days (Figure 4B). Primary root elongation of *35S*-*APUM5* was also hypersensitive on the 1/2 MS plate containing 300 mM mannitol (Additional file 5B). These results suggest that APUM5 may negatively contribute to salt and dehydration stress tolerance.

Fully germinated 3-day-old seedlings in normal 1/2 MS medium were transferred to plates supplemented with 150 mM NaCl or 400 mM mannitol to further assess the effect of salt or dehydration on growth of *APUM5* transgenic seedlings, and primary root elongation was monitored after 7 days. Primary root elongation of the *APUM5* transgenic plants was similar to that of the Col-0 plants under normal conditions (Figure 4C). However, primary root length of *APUM5*-RNAi plants was longer than that of Col-0 plants under the 150 mM NaCl supplement condition, whereas that of *35S*-*APUM5* transgenic plants was shorter (Figure 4D). This phenotype is thus concentration-dependent effect in root elongation of *35S-APUM5* plants. In contrast, *APUM5*-RNAi transgenic plants showed enhanced primary root growth under the salinity stress condition (Figure 4D). The primary root length of *APUM5-*RNAi transgenic plants was 14–15% longer compared with that of Col-0 plants when the plants were grown on plates supplemented with 400 mM mannitol, whereas *35S*-*APUM5* plants showed reduced primary root growth of 16–17% compared to that of Col-0 plants (Figure 4E).

We assessed whether germination and primary root growth were affected by an exogenous ABA application. The germination rate of *35S*-*APUM5* transgenic plants decreased about 39% and 49% compared with that of wild-type and *APUM5*-RNAi plants following 0.5 μM and 0.7 μM ABA treatments, respectively (Additional file 6A). Primary root length of *35S*-*APUM5* plants was shorter than that of the wild-type and *APUM5*-RNAi plants following ABA treatment (Additional file 6B). These results show that *APUM5*-overexpressing plants are more hypersensitive to dehydration or salt stress, suggesting that APUM5 might regulate the abiotic stress response.

**Figure 5:**
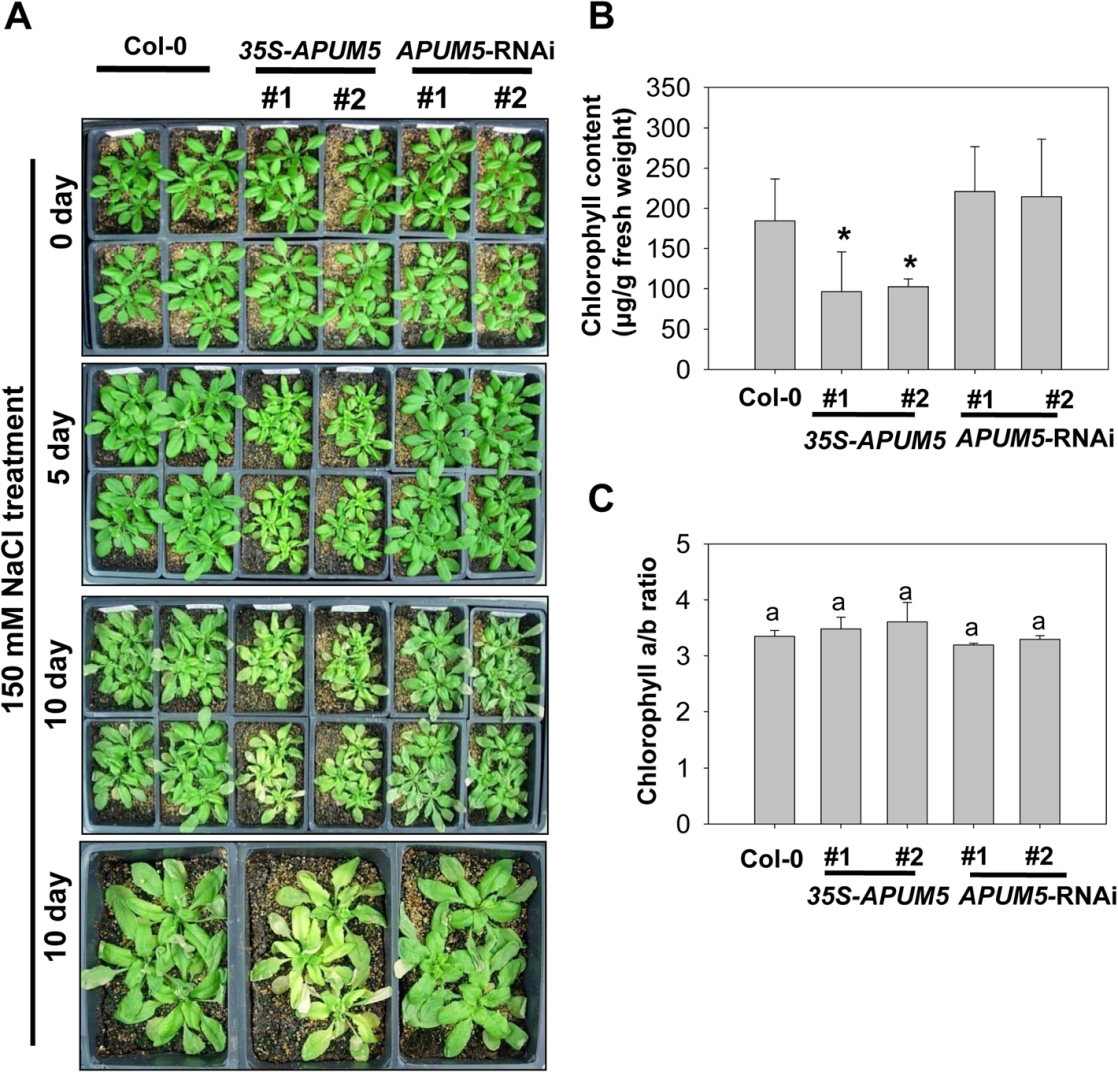
**Analysis of salt sensitivity in wild type and *APUM5 *transgenic plants in soil. (A)** Photographs show plants treated with 150 mM NaCl at the indicated time points. Four-week-old Col-0, *35S*-*APUM5*, and *APUM5*-RNAi plants were treated with 150 mM NaCl. **(B)** Chlorophyll contents of salt-treated plants on day 10. Pigments were extracted from the salt-treated plant leaves. Data are mean values of six independent experiments. **(C)** Chlorophyll a/b ratio of salt-treated plant leaves. Error bars represent ± SD (Student’s *t*-test; *P < 0.01).

APUM5 seems to be directly involved in the osmotic stress response. Phenotypes of wild type and *APUM5* transgenic plants treated with salt were evaluated in soil to investigate the possible role of APUM5 in the salt stress response. A high-salinity treatment resulted in symptoms on Col-0 leaves such as chlorosis, leaf burn, and senescence as well as a decrease in leaf area compared with those in non-stressed plants [29]. Wild-type and *APUM5* transgenic plants showed similar normal growth at the vegetative stage. However, *35S*-*APUM5* transgenic plants exhibited a slightly more shrinking phenotype compared with Col-0 and *APUM5*-RNAi plants when irrigated with 150 mM NaCl for 5 days (Figure 5A). *35S*-*APUM5* transgenic plants showed significantly enhanced chlorosis, leaf burn, and reduced leaf area at 10 days, compared with that in Col-0 and *APUM5*-RNAi plants (Figure 5A). To further analyze the effect of salt stress, chlorophyll contents and chlorophyll a/b ratio were measured because chlorosis was enhanced in *APUM5*-overexressing plants. Chlorophyll content decreased but the chlorophyll a/b ratio remained unchanged in *35S*-*APUM5* transgenic plants compared to those in Col-0 and *APUM5*-RNAi plants (Figure 5B and C). Taken together, these results indicate that *APUM5*-overexpressing plants exhibit hypersensitivity to salt stress at the vegetative and primary root elongation stage, suggesting that APUM5 may act as a negative regulator when plants are subjected to salt stress.

### *APUM5*-overexpressing plants are more hypersensitive whereas *APUM5*-knockdown plants are more tolerant to drought stress compared to wild-type plants in soil

**Figure 6:**
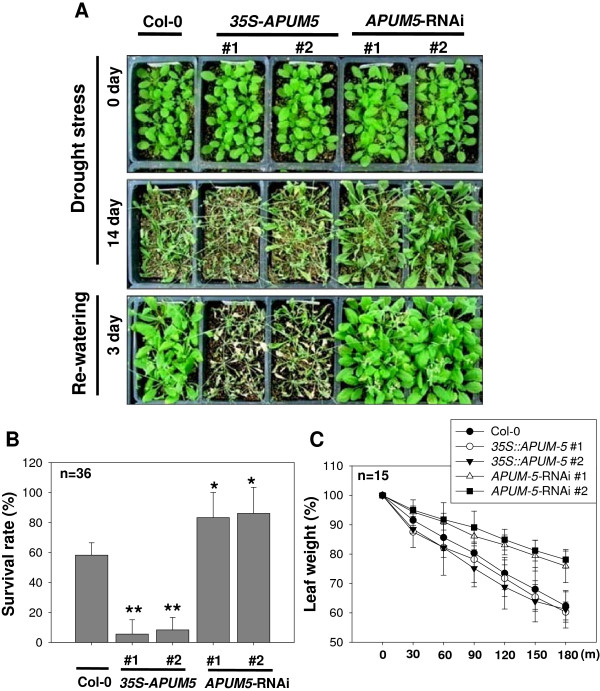
**Drought sensitivity and water loss analysis of wild type and *APUM5 *transgenic plants. (A)** The photographs show increased drought sensitivity of *35S*-*APUM5* transgenic plants and decreased sensitivity of *APUM5*-RNAi plants. Irrigation was stopped in 4-week-old plants for 14 days and then they were re-watered for 3 days afterwards. **(B)** Percentage survival of wild type and *APUM5* transgenic (*35S*-*APUM5* and *APUM5*-RNAi) plants. Each group of 30–40 plants was averaged to measure survival rate (Student’s *t*-test; *P < 0.01, **P < 0.001). **(C)** Water loss measurements in detached rosette leaves of wild-type and *APUM5* transgenic plants after the drought stress treatment. Detached rosette leaves were measured and monitored for loss of fresh weight at the indicated time points. Data are mean values of three independent experiments (n = 15).

We further investigated whether *APUM5* transgenic plants showed an altered phenotype to drought tolerance. Both Col-0 and *35S*-*APUM5* plants became severely wilted when water was withheld from soil for 14 days (Figure 6A). However, down-regulation of *APUM5* by *APUM5*-RNAi resulted in the enhanced tolerant phenotype at the same stage compared with that of Col-0 (Figure 6A). Survival rate was examined after re-watering the 14 day-water-withheld plants. Approximately 77% of the *APUM5*-RNAi plants survived, whereas 11–13% of the *35S*-*APUM5* and 58% of the Col-0 plants survived (Figure 6B). Additionally, water loss rate of the wild-type and *APUM5* transgenic plants was measured in detached leaves. Wild-type plants exhibited similar weight loss of detached leaves as *APUM5*-RNAi plants, whereas *35S*-*APUM5* transgenic plants had highly enhanced water loss rates (Figure 6C). Thus, similar to the salt stress results, *APUM5*-overexpressing plants exhibited more drought sensitivity, whereas repressing of *APUM5* expression led to more drought tolerance compared with that of wild-type plants in soil.

### Altered transcription levels of abiotic stress-responsive genes in *APUM5*-overexpressing plants under drought stress

**Figure 7:**
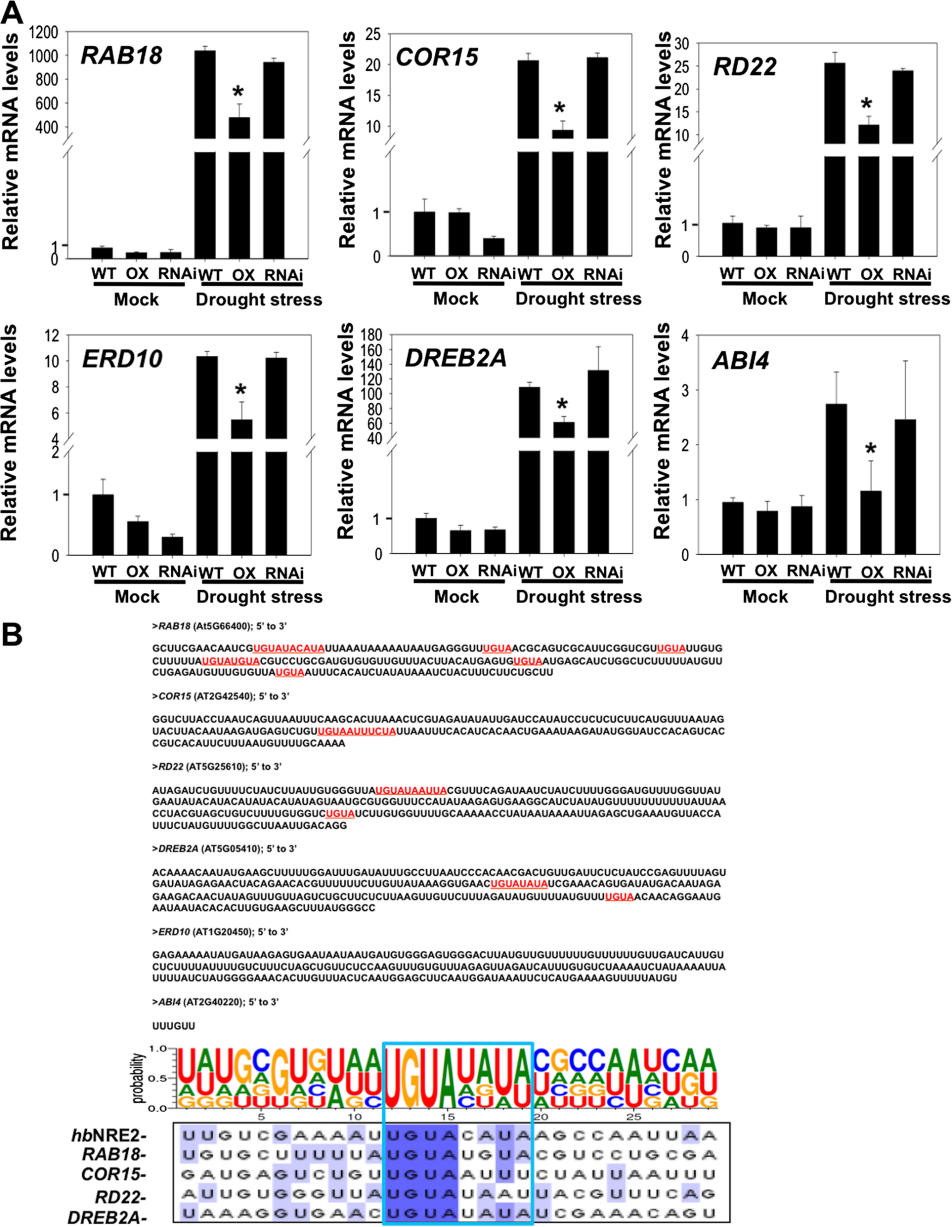
**Real-time qRT-PCR analysis of ABA-responsive genes in wild type and *APUM5 *transgenic plants under drought stress. (A)** Expression of the ABA-regulated genes in wild-type and *APUM5* transgenic plants under drought stress. mRNA levels were determined by qRT-PCR using total RNAs isolated from control and 6 h drought-stressed detached rosette leaves of wild-type and *APUM5* transgenic plants. Data are mean values of three independent experiments (Student’s *t*-test; *P < 0.01). **(B)** Sequence analysis of the putative APUM5 binding sites in the 3′ UTRs of abiotic stress-related genes. The putative binding sequence including ‘UGUA’ is highlighted. *hb*NRE2 was used as a conserved Pumilio RNA binding motif. The ABA-responsive genes down-regulated in the *35S*-*APUM5* transgenic plants under drought stress were analyzed at the 3′ UTR sequence data base of The *Arabidopsis* Information Resource (TAIR) and UTRdb (http://utrdb.ba.itb.cnr.it/). The motif was used to search for *Drosophila hb*NRE2-related sequences and reconstructed using sequence alignment (CLUSTALW) and WebLogo software.

A gene expression analysis of various genes was performed by qRT-PCR to examine whether the enhanced sensitivity of *35S*-*APUM5* transgenic plants to salt and drought stresses was accompanied by changed transcription levels of abiotic stress-responsive genes. The transcription levels of some abiotic stress-responsive genes decreased in *35S*-*APUM5* transgenic plants under the drought stress condition compared to those in wild type plants (Figure 7A). The putative APUM5 binding site was searched in the 3′ UTR region of abiotic stress-responsive genes to investigate the possible role of APUM5 as a post-transcriptional repressor. We conducted this search because mammalian Pumilio proteins directly interact with target transcripts containing the conserved ‘UGUA’ tetranucleotide motif [9,15,17]. Some of 3′ UTRs of abiotic stress-responsive genes contained the conserved ‘UGUA’ tetranucleotide motif (Figure 7B and Additional file 7B). The 3′ UTRs of significantly downregulated genes in *35S*-*APUM5* transgenic plants by drought stress were re-analyzed and compared with the *Drosophila hb*NRE2 sequence. The ‘UGUA’ tetranucleotide motif of the 3′ UTRs was highly conserved among the most abiotic stress-responsive genes and *hb*NRE2 (Figure 7B). *ERD10* and *ABI4* transcript levels also decreased about 52% and 42% in *APUM5*-overexpressing plants compared with wild-type plants upon drought stress, respectively (Figure 7A). However, *ERD10* and *ABI4* did not have the ‘UGUA’ motif in the 3′ UTR region (Figure 7B). In contrast, *KIN1*, *AtMYB6*, *AAO3*, and *RD29B* did not contain the ‘UGUA’ tetranucleotide motif and their transcript levels did not change significantly in *35S*-*APUM5* plants upon drought stress (Additional file 7). *APUM5*-RNAi plants did not exhibit a dramatic alteration in ABA-response genes expression partly because *APUM5*-RNAi transgenic plants showed only 50% silencing levels of the *APUM5* gene and the effect may be similar to that of the heterozygote mutant (Huh *et al.*, 2013). These results suggest that transcripts of some abiotic stress-responsive genes could be negatively regulated by binding of the APUM5 protein in their 3′ UTR regions.

### APUM5-PHD binds to the 3′ UTR motifs of abiotic stress-responsive putative target genes

**Figure 8:**
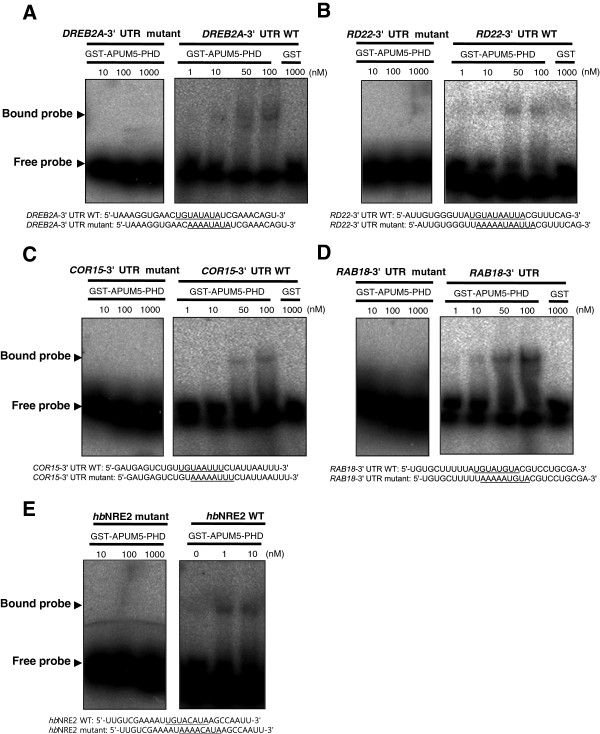
**Gel mobility shift assay of the recombinant containing APUM5-PHD for 3′ UTR motifs of abiotic stress-responsive genes. (A-D)**. Indicated ^32^P-labeled RNA probes were incubated with recombinant GST-APUM5-PHD (1, 10, 50, and 100 nM) for 30 min in 20 μl RNA binding buffer at room temperature. RNA-protein complexes were separated on a 5% native gel and analyzed by autoradiography. GST protein (1000 nM) was used as a negative control. **(E) **^32^P-labeled *hb*NRE2 RNA probes were incubated with recombinant GST-APUM5-PHD (1 and 10 nM) and used as a positive binding control.

An electrophoretic mobility shift assay (EMSA) was performed to determine if the APUM5-PHD binds to the putative target RNAs. Genes that were highly down-regulated by APUM5 and had a ‘UGUA’ binding motif were selected. ^32^P-labeled synthetic 30 nucleotide RNAs along with ‘UGUA’ core sequence mutants were incubated with recombinant GST-APUM5-PHD protein. GST was used as a negative control. The EMSA results revealed that GST-APUM5-PHD bound effectively to *DREB2A*, *RD22*, *COR15*, and *RAB18* but not to mutant RNAs, whereas the GST protein did not interact with these RNAs (Figure 8A–D). Furthermore, APUM5-PHD also showed strong binding affinity for *hb*NRE2 RNA (Figure 8E). APUM5 recognized the 8–10 nucleotide ‘UGUA’ core motifs. These results indicate that APUM5 binding affinity might be flexible for target binding motif recognition and this flexibility could contribute to multi-regulation of abiotic stress-responsive genes by destabilizing target mRNAs. This result confirmed that the *Arabidopsis* APUM5 protein has RNA binding activity and that the binding is important for regulating putative target 3′ UTRs.

### APUM5 negatively regulates the *RD22* and *RAB18* 3′ UTR reporters

**Figure 9:**
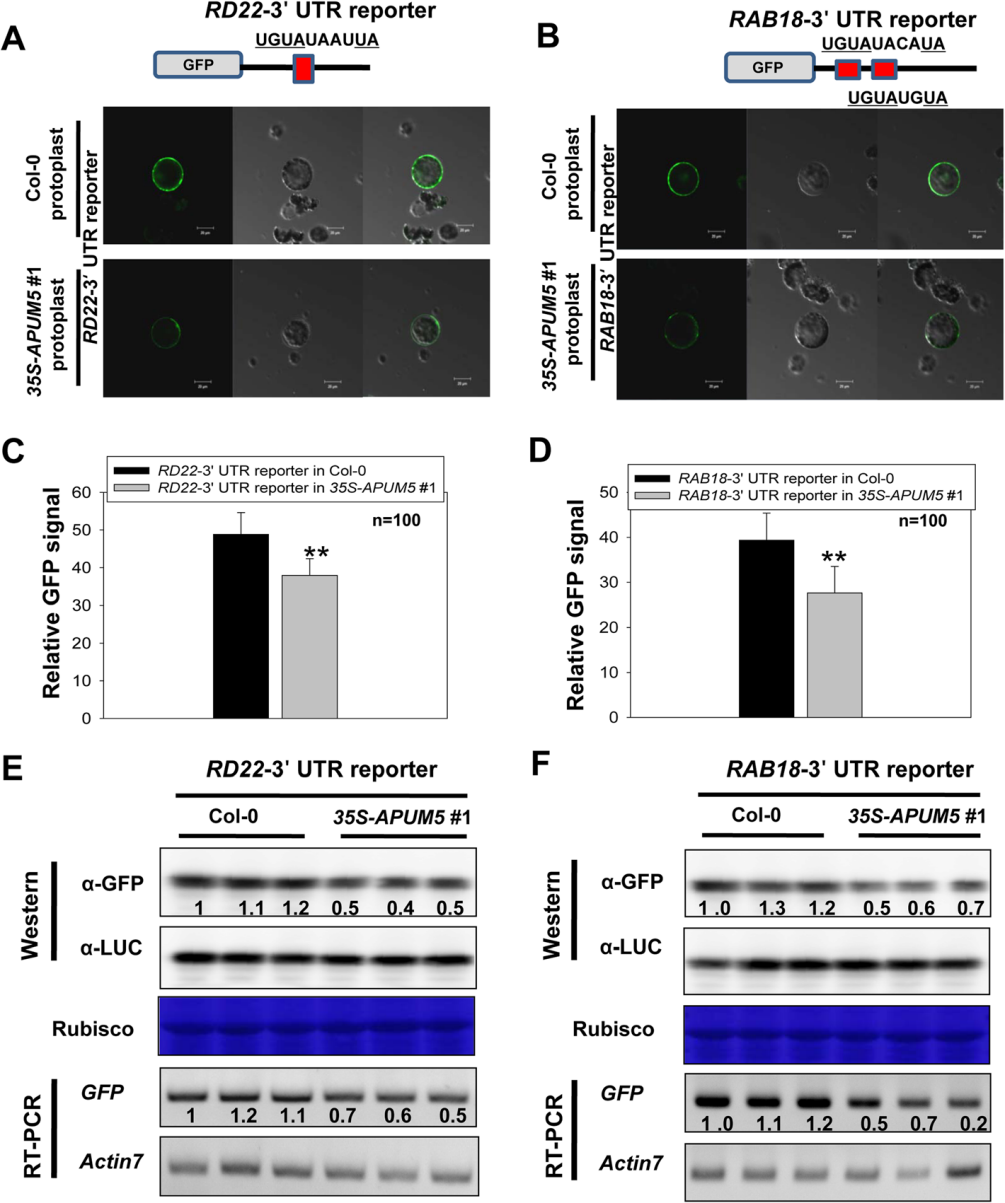
**APUM5 negatively regulates the 3′ UTRs of ABA-responsive genes in the reporter assay. (A and B)** Protoplasts of Col-0 and *35S*-*APUM5* transgenic plants were transformed with the *RD22*-3′ UTR or *RAB18*-3′ UTR reporter construct by PEG-mediated transformation. GFP signals were detected by LSM 700 confocal microscopy under identical conditions. **(C, D)** GFP signal intensity of the *RD22*-3′ UTR and *RAB18*-3′ UTR reporter were analyzed with Zen software of the LSM 700 confocal microscope and ImageJ software (rsbweb.nih.gov/ij/). Error bars represent ± SD (Student’s *t*-test; **P < 0.001). **(E, F)** Western blot and RT-PCR analysis were performed with protoplasts of Col-0 and *35S*-*APUM5* transgenic plants transformed with the *RD22*-3′ UTR or *RAB18*-3′ UTR reporter construct. Protein and RNA samples were obtained from four-independent experiments and extracted. Rubisco was used as the protein loading control and *AtActin7* was used as the internal control. The numbers designate relative intensity.

We found that the APUM5-PHD protein directly bound to 3′ UTRs of ABA-responsive genes *in vitro*. This phenomenon might explain that APUM5 negatively regulates ABA-responsive genes via binding to target 3′ UTRs. We made reporter constructs with 3′ UTRs of ABA-responsive genes and expressed reporter constructs in Col-0 and *35S*-*APUM5* transgenic protoplasts to identify the function of this binding. The *RD22*-3′ UTR reporter normally expressed GFP signals in Col-0 protoplasts, whereas the *RD22*-3′ UTR reporter showed reduced GFP signals in *35S*-*APUM5* transgenic protoplasts (Figure 9A). Next, these GFP signals were quantified by confocal LSM700 microscopy and ImageJ software. The signal intensity of the *RD22*-3′ UTR reporter in *35S*-*APUM5* transgenic protoplasts decreased approximately 20% compared with that in Col-0 protoplasts (Figure 9C). Western blot with a GFP antibody and RT-PCR analyses were performed to further confirm these data. Both the GFP protein and RNA levels of the *RD22*-3′ UTR reporter in *35S*-*APUM5* transgenic protoplasts decreased compared with the levels in Col-0 protoplasts (Figure 9E). We also performed the reporter assay with the *RAB18*-3′ UTR reporter using a similar procedure. The *RAB18*-3′ UTR reporter in *35S*-*APUM5* transgenic protoplasts showed about a 45% reduction in GFP signal intensity compared with that in Col-0 protoplasts (Figure 9B and D). In the Western blot and RT-PCR analyses, GFP protein and RNA levels decreased slightly in *35S*-*APUM5* transgenic protoplasts compared with those in Col-0 protoplasts (Figure 9F).

Previous results showed that APUM5-PHD also bound to the *DREB2A* and *COR15* 3′ UTRs (Figure 8A and C), and these putative target RNAs also contained the PHD binding RNA motif. Thus, we expected that these reporters would also exhibit a decrease of the GFP signal in *35S*-*APUM5* transgenic protoplasts. However, the GFP protein and RNA levels of the *DREB2A* and *COR15* 3′ UTR reporters were not affected in *35S*-*APUM5* transgenic protoplasts compared with Col-0 protoplasts (Additional file 8). These data indicate that APUM5 negatively regulates some ABA-responsive genes by binding to 3′ UTR post-transcriptionally. Furthermore, RNA binding motifs may be very flexible and other RNA residues in addition to the ‘UGUA’ core motif or RNA structure could be important for the *in vivo* APUM5 binding system.

## Discussion

*Arabidopsis* and rice have multiple Puf members [11]. However, the function of plant Pufs is poorly understood. We performed an *APUM5* gene expression analysis using a bioinformatics tool to functionally characterize *APUM5*, which is related to the biotic and abiotic stress responses. *APUM5* showed increased expression patterns following exposure to biotic and abiotic stressors. We showed here that the *Arabidopsis* Puf protein, APUM5, negatively regulated some abiotic stress responsive genes and could be involved in the osmotic and drought stress response, although APUM5 was not involved in susceptibility or resistance to bacterial and fungal pathogen infection (Figure 1C and D). However, *APUM5* is a pathogen responsive gene upon bacterial and fungal pathogen infection (Figure 1A and B). APUM5 function may still be related to defense response regulation, although we did not show any definitive results for specific bacterial or fungal infections. Similar to animal Puf proteins, APUM5 may regulate diverse target genes and be regulated by defense-related factors such as MPK4 (Additional file 1).

ABA-mediated plant immunity has been highlighted in terms of virus-host interaction. When *Tobacco mosaic virus* (TMV-cg) infects ABA mutant plants, TMV-cg accumulation increases in the systemic region and TMV-cg could repress ABA signaling by repressing WRKY8 [30]. Furthermore, CMV 2b, a silencing suppressor, interferes with ABA-mediated signaling, and transgenic plants that overexpress CMV 2b exhibit enhanced tolerance to drought stress [31]. In contrast, overexpression of *APUM5* led to more resistance to CMV infection and slightly diminished *Turnip mosaic virus* accumulation, although APUM5 may negatively regulate some of the ABA responsive genes [22]. This phenotype demonstrates that some plant viruses could negatively regulate ABA signaling by regulating host proteins. However, APUM5 defense function against CMV infection might not be directly connected with regulation of some ABA responsive genes.

The *APUM5*-overexpressing transgenic plants exhibited reduced expression of some abiotic-stress response genes (Figure 7A). Furthermore, the core Puf binding motif in the putative target genes is found at the 3′ UTR (Figure 7B), indicating that APUM5 is likely to control these putative target mRNAs by binding to the 3′ UTR. Puf3, a yeast Puf homolog protein, binds to the 3′ UTR of *COX17* mRNA and regulates *COX17* mRNA by enhancing RNA degradation [32]. Interestingly, the CMV 3′ UTR is not polyadenylated but contains the tRNA-like structure (TLS). APUM5 does not affect the RNA level of the CMV 3′ UTR reporter [22]. Thus, the function of APUM5 during abiotic stress could be different from the plant defense mechanism against CMV infection because APUM5 slightly affected reporter mRNA level (Figure 9E and F). This result indicates that APUM5 function could possibly be associated with a putative deadenylase complex such as the mammalian Puf-deadenylase complex [17], although we showed that the putative Ccr4-Pop2p-NOT mRNA deadenylase complex is not a APUM5 binding partner [23].

The putative APUM5 targets, *DREB2A*, *RD22*, *COR15*, and *RAB18* contained ‘UGUA’ core binding motifs in the 3′ UTRs when compared with the *hb*NRE2 motif. One of them, *RAB18* 3′ UTR motif showed stronger binding affinity to APUM5-PHD protein compared to other motifs by binding even at 1 nM concentration (Figure 8). This that APUM5-PHD directly bound to these motifs *in vitro*, suggesting that the ‘UGUA’ core motif confers RNA-binding activity of Puf proteins but other RNA residues or secondary structures of the core motif are also important for Puf protein binding affinity *in vivo*[33]. In our results, *ABI4* gene expression level in *35S*-*APUM5* transgenic plants decreased upon drought stress (Figure 7A). However, it did not contain the ‘UGUA’ core motif in its 3′ UTR sequence (Figure 7B), indicating that APUM5 might directly and indirectly regulate targets negatively.

ABA plays a key role in osmotic tolerance by regulating ABA-responsive transcription factors such as ABI3 and ABI5 [34]. ABA also affects seed germination [35]. *APUM5* was highly expressed under the mannitol, NaCl, and exogenous ABA treatments (Figure 2). The seed germination and root growth experiments exhibited *35S-APUM5* transgenic plants were hypersensitive while *APUM5*-RNAi plants were insensitive compared to Col-0 wild-type plants under osmotic stress and exogenous ABA treatment of various concentrations (Figure 4 and Additional file 6). Interestingly, *35S*-*APUM5* transgenic plants were hypersensitive to salinity and drought stress in soil (Figures 5 and 6) although most ABA-sensitive mutants show salinity or drought tolerance phenotypes in soil [36,37]. Furthermore, some mutants exhibit development-dependent phenotypes to abiotic stress treatments. For example, *AREB1*, *AREB2*, and *ABF3* mutants do not exhibit ABA sensitive phenotypes in single, double, or triple mutants during germination. However, these mutants show the ABA-sensitive phenotype during primary root growth [38]. These data indicate that some mutants exhibit development-dependent phenotypes to exogenous ABA application. In contrast, some *Arabidopsis* proteins control the ABA-dependent and ABA-independent pathways to the abiotic stress response [37,39]. For example, OSM1, a SNARE superfamily protein, is important to osmotic stress tolerance and the *osm1* mutant exhibits a hypersensitive phenotype during seed germination under an osmotic stress condition. The *osm1* mutant plants also show increased sensitivity to salt and soil desiccation [39].

An examination of tissue-specific activities of *APUM5pro*-*GUS* transgenic plants revealed high-level expression in roots under normal condition (Figure 3A). Furthermore, the *APUM5pro*-*GUS* activity assay showed enhanced levels under diverse abiotic stressors in hydathodal cells and trichomes (Figure 3B and C). Hydathodal cells are involved in secreting water [40,41], and trichomes generally protect leaves by insulating them from heat, salt, insects, and water loss [42]. These expression patterns indicate that APUM5 may be involved in this tissue-specific regulation and affect the salinity and drought stress condition, although *APUM5* was not expressed in guard cells (Figure 3C).

## Conclusions

Our results provide evidence that *Arabidopsis* Pumilio proteins have functionally conserved RNA binding domain and activity. We found that *APUM5* is a defense-responsive gene and negatively regulated the *RD22*-3′ UTR and *RAB18*-3′ UTR reporters at the mRNA and protein levels. This negative regulation of APUM5 could be connected to a more sensitive *35S-APUM5* transgenic plant phenotype under abiotic stress. These data suggest that the APUM5 protein could have dual functions in both transcriptional regulation and translational control by binding with target 3′ UTR motifs and may have diverse functions during biotic stress, abiotic stress, and development. But still we do not know the real targets of APUM5 *in vivo*. To understand the Puf protein functions further, the real targets of APUM5 should be identified by immunoprecipitation of mRNA-APUM5 complexes.

## Methods

### Plant material, growth conditions, and transgenic plants

*APUM5* transgenic plants were prepared as described previously [22]. *A. thaliana* Col-0 and *APUM5* transgenic plants were grown in a 16 h light/8 h dark photoperiod at 23°C in soil.

### Pathogen inoculation

*Pst* DC3000 was cultured in King’s B medium and resuspended in 10 mM MgCl_2_ for bacterial pathogen inoculation. Leaves of 4-week-old Col-0, *APUM5* transgenic, and *NahG* plants were syringe infiltrated with *Pst* DC3000 suspensions (OD_600_ = 0.0001 in 10 mM MgCl_2_). Leaf discs were collected from Col-0 and other mutant leaves at 0 and 3 days post-inoculation (dpi) to detect bacterial growth. Bacterial counts on leaf discs were measured after 28°C incubation for 2 days as described previously [43]. *A. brassicicola* strain KACC40036 was grown on potato dextrose agar for the fungal pathogen disease assay (Difco Scientific, Detroit, MI, USA). A spore suspension (5 × 10^4^ spores/mL in potato dextrose media) was dropped on the detached leaves of Col-0 and *APUM5* transgenic plants and was kept under high humidity. The spore population was counted at 5 dpi [43].

### Germination rate and root growth assays

Surface-sterilized seeds of *Arabidopsis* Col-0, *APUM5*-RNAi, and *35S*-*APUM5* plants were grown on plates containing 1/2 Murashige and Skoog Medium Basal Salt Mixture (Duchefa, Haarlem, The Netherlands), 1.5% sucrose, and 0.8% phytoagar with or without various concentrations of NaCl, mannitol, and ABA at 22°C under a long day condition to determine the effect of osmotic stress and ABA on germination. The percentage of germinated seeds was scored daily in three independent experiments (60–70 seeds per experiment).

A root growth assay was carried out by transferring 3-day-old seedlings to the minimal medium supplemented with different concentrations of NaCl, mannitol, and ABA to determine the effect of osmotic stress and ABA on root growth. After 7 or 10 days, root growth was measured in three independent experiments (30–40 seedlings per experiment).

### Manipulation of RNA and quantitative gene expression analysis under the various stress conditions

Ten-day-old seedlings were submerged in ddH_2_O supplemented with the indicated concentrations of mannitol, NaCl, or ABA for the gene expression analysis. Rosette leaves of 4-week-old plants were dehydrated on Whatman paper at 25°C under light for the indicated time period to test the effect of desiccation. Four-week-old plants were syringe infiltrated with a bacterial suspension (OD_600_ = 0.001 in 10 mM MgCl_2_) of virulent *Pst* DC3000 for the gene expression analysis under the biotic stressors. *Arabidopsis* plants were inoculated with *A. brassicicola* by spraying a spore suspension (5 × 10^5^ spores/mL) in potato dextrose media for the gene expression pattern analyses. These samples were harvested at the indicated time points, and total RNA was isolated by the modified hot-phenol method [44] and reverse-transcribed with MMLV Reverse Transcriptase (Promega, Madison, WI, USA). Real-time PCR was performed according to the instructions provided by the LightCycler Real-Time PCR Systems (Roche, Mannheim, Germany). The primers used for real-time PCR reactions are listed in Additional file 9.

### Histological assays

For histochemical analysis of the GUS activity, seedlings or plants from the T3 populations under the normal or stressed conditions were vacuum-infiltrated with *Agrobacterium* carrying the constructs in X-Gluc staining solution (0.5 M sodium phosphate buffer, pH7.0, 10% Triton X-100, 0.1 M K_3_Fe(CN)_6_, 0.1 M K_4_Fe(CN)_6_, 2 mM X-GlcA), incubated at 37°C overnight, and transferred into 70% (v/v) ethanol to remove chlorophyll [45]. GUS activity was observed under an Axioplan 2 imaging microscope (Carl Zeiss, Jena, Germany) and COOLPIX 5200 digital camera (Nikon, Osaka, Japan).

### High-salinity and drought stress treatment in soil

Four-week-old Col-0, *APUM5*-RNAi, and *35S*-*APUM5* plants were irrigated with 150 mM NaCl every 3 days and allowed to grow for 3 weeks to test the effect of high-salinity stress. For the drought stress treatment, 4-week-old stage plants grown in soil were deprived of water for 14 days. Then, these plants were provided water. Surviving plants were counted at 3 days after re-watering. Three independent measurements of 30–40 plants were averaged.

### Gel mobility shift assay

Synthetic RNA oligonucleotides were obtained from Bio Basic Inc. (Markham, ONT, Canada). An RNA probe was radiolabeled with ^32^P using T4 polynucleotide kinase (New England Biolabs, Ipswich, MA, USA) and incubated with recombinant GST-APUM5-PHD for 30 min in a binding solution (20 mM Tris at pH 7.4, 50 mM KCl, 3 mM MgCl_2_, 2 mM dithiothreitol, 5% glycerol) at room temperature. Samples were separated on a 4% native gel containing 5% glycerol in 0.5 × TBE buffer, and subjected to autoradiography.

### Reporter assay

To generate reporter constructs, the full *RD22*, *RAB18*, *DREB2A*, and *COR15*-3′ UTR sequences were isolated and ligated into the N-terminus of the modified 326-GFP3G vector [22]. Protoplasts of Col-0 and *APUM5* transgenic plants were isolated, and reporter constructs were introduced into the protoplasts by PEG-mediated transformation [46], with several modifications. GFP signal intensity was quantified by Zen software for the LSM 700 confocal microscope and ImageJ software (rsbweb.nih.gov/ij/).

## Competing interests

The authors declare that they have no competing interests.

## Authors’ contributions

SUH and KHP conceived the study, designed the experiments and drafted the manuscript. SUH performed the experiments. SUH and KHP read and approved the final manuscript.

## Supplementary Material

Additional file 1 **Expression analysis of *APUM5 *in *mpk4-1 *compared to L*er *control plants.** Total RNAs were extracted from 3-week old plants and mRNA levels were determined by qRT-PCR analysis. Error bars represent ± SD (n = 3). (Student’s *t*-test; ***P < 0.0001). Click here for file

Additional file 2 **GUS activity analysis of *APUM5pro*-*GUS *transgenic plants at the flowering and silique development stages.***APUM5* promoter activity was determined by histochemical GUS staining at the flower stage. **(A)** Cauline leaves and flowers. **(B)** Flowers. **(C)** Silique. Arrows indicate the end regions of the silique. Click here for file

Additional file 3 ***Cis*-element analysis of the *APUM5 *promoter.** A 1.3-kb promoter region was isolated using Athena analysis tools (http://www.bioinformatics2.wsu.edu/Athena) to analyze *APUM5*promoter sequences. Click here for file

Additional file 4 **Germination rate analysis of *APUM5 *** **transgenic plants in 1/2 MS medium plates (n = 150).** Click here for file

Additional file 5 **Salt and mannitol sensitivity of wild-type and *APUM5 *transgenic plants. (A)** The effect of 100 mM NaCl on primary root elongation. **(B)** The effect of 300 mM mannitol on primary root elongation. Seeds were germinated for 3 days on 1/2 MS medium, and the seedlings were transferred (n = 30, triplicates) to 1/2 MS containing 100 mM NaCl or 300 mM mannitol. Primary root length was measured at 10 days after transfer. Click here for file

Additional file 6 **ABA effect on seed germination and primary root growth in wild-type and *APUM5 *transgenic plants. (A)** The effect of ABA on seed germination. Seeds were germinated and grown on 1/2 MS medium containing 0.5 and 0.7 μM ABA for 7 days, and seedlings with green cotyledons were counted (n > 60, triplicates). **(B)** The effect of ABA on primary root elongation. Seeds were germinated for 3 days on 1/2 MS medium, and the seedlings were transferred (n = 30, triplicates) to 1/2 MS medium containing 2 and 5 μM ABA, respectively. Primary root length was measured at 10 days after transfer. Click here for file

Additional file 7 **Expression analysis of some abiotic stress-responsive genes that do not have a putative APUM5 target site in *APUM5 *transgenic plants upon drought stress. (A)** mRNA levels were determined by qRT-PCR analysis using total RNAs isolated from control and 6 h drought-stressed detached leaves. Error bars represent ± SD (n = 3). **(B)** The 3′ UTRs of abiotic stress-responsive genes were analyzed using the *Arabidopsis* Information Resource (ftp://ftp.arabidopsis.org/home/tair/Genes/TAIR10_genome_release/) and the 3′ UTR sequence database UTRdb (http://utrdb.ba.itb.cnr.it/). Red indicates ‘UGUA’ tetranucleotide Puf target motif. Click here for file

Additional file 8 **Reporter assay. (A, B)** Protoplasts of Col-0 and *35S*-*APUM5* transgenic plants were transformed with the *DREB2A*-3′ UTR reporter or the *COR15*-3′ UTR construct by PEG-mediated transformation. GFP signals were detected by LSM 700 confocal microscopy under identical conditions. **(C, D)** GFP signal intensity of the *DREB2A*-3′ UTR and *COR15*-3′ UTR reporters was quantified with Zen software of the LSM 700 confocal microscope and ImageJ software (rsbweb.nih.gov/ij/). Error bars represent ± SD. **(E, F)** Western blot and RT-PCR analyses were performed with protoplasts of Col-0 and *35S*-*APUM5* transgenic plants transformed with the *DREB2A-*3′ UTR reporter or the *COR15*-3′ UTR construct. Protein and RNA samples were extracted from four-independent experiments. Rubisco was used as a protein loading control and *AtActin7* was used as the internal control. Click here for file

Additional file 9 Coupled RT and qRT-PCR primers used for expression analysis of abiotic stress-related genes. Click here for file
