# Supplementary figures and images for: APUM5, encoding a Pumilio RNA binding protein, negatively regulates abiotic stress responsive gene expression
Source: BMC Plant Biol. 2014 Mar 25;14:75. doi: 10.1186/1471-2229-14-75 (PMC3986970; doi:10.1186/1471-2229-14-75)

**A****150 mM NaCl**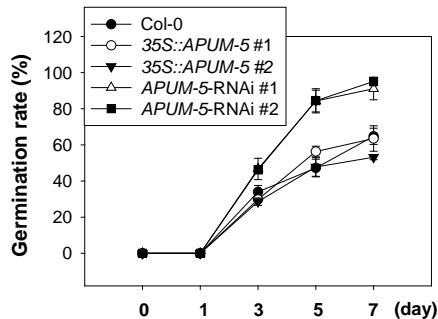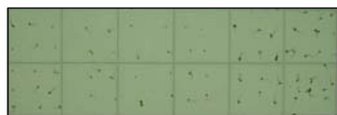**150 mM NaCl (7 day)****B****400 mM mannitol**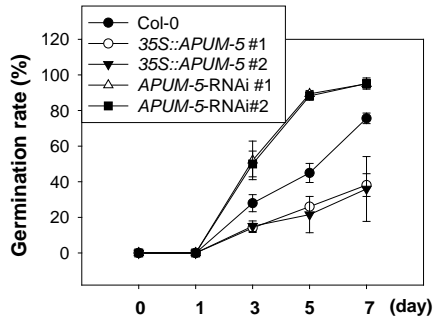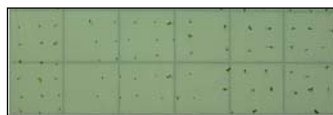**400 mM mannitol (7 day)****C**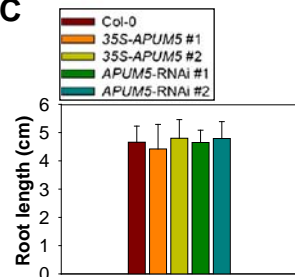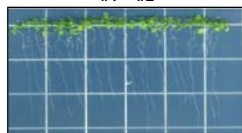**1/2MS plate (7 day)****D**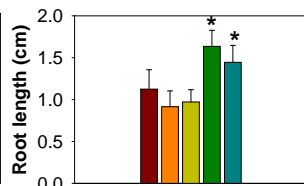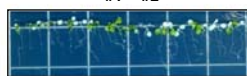**150 mM NaCl (7 day)****E**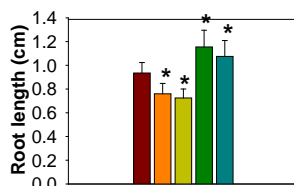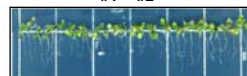**400 mM mannitol (7 day)**

Supplement: Additional file 1 — Expression analysis of APUM5 in mpk4-1 compared to Ler control plants. Total RNAs were extracted from 3-week old plants and mRNA levels were determined by qRT-PCR analysis. Error bars represent ± SD (n = 3). (Student’s t-test; ***P < 0.0001). [file 1471-2229-14-75-S1.pdf]

Additional file 2

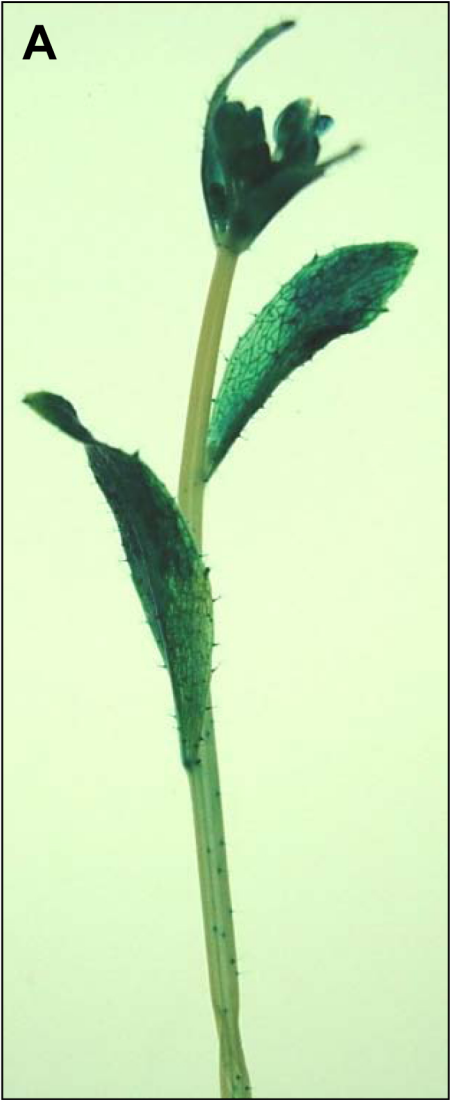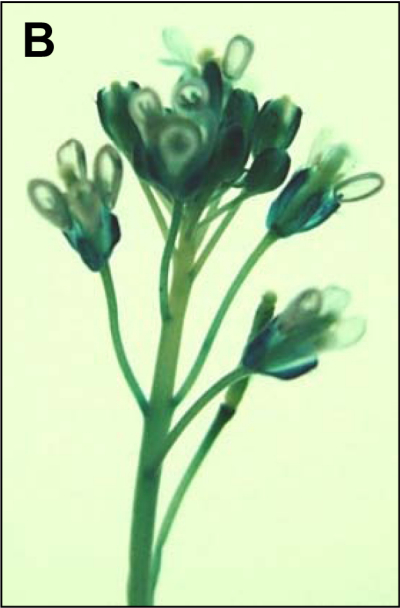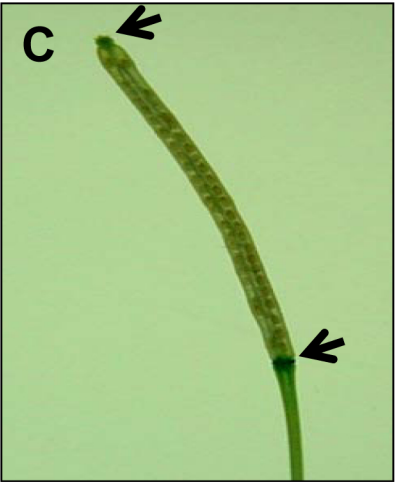

Supplement: Additional file 2 — GUS activity analysis of APUM5pro-GUS transgenic plants at the flowering and silique development stages.APUM5 promoter activity was determined by histochemical GUS staining at the flower stage. (A) Cauline leaves and flowers. (B) Flowers. (C) Silique. Arrows indicate the end regions of the silique. [file 1471-2229-14-75-S2.pdf]

*APUM5* promoter

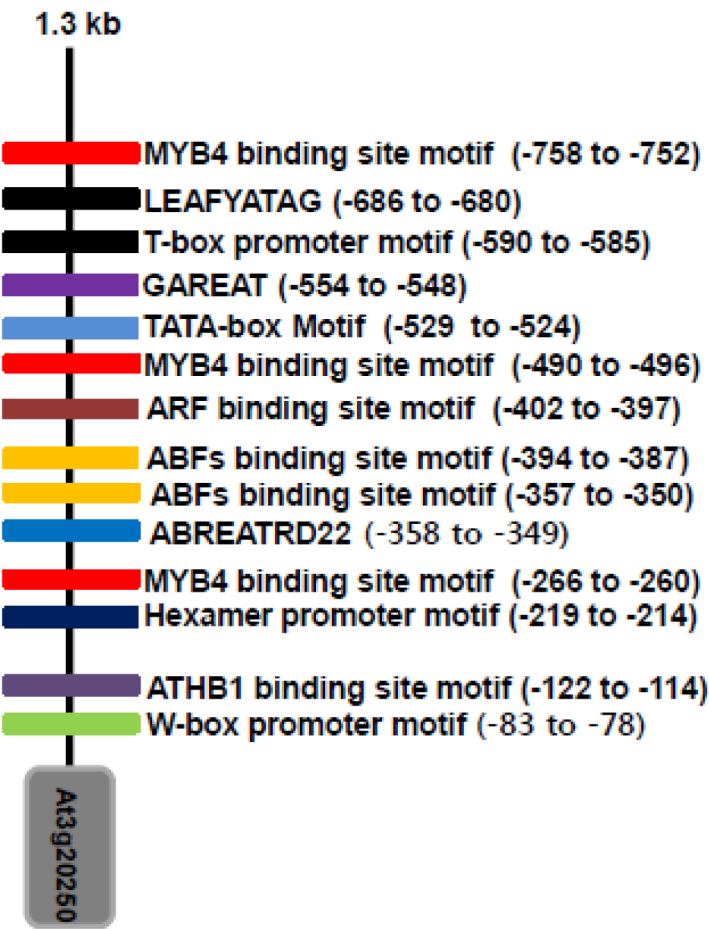

Supplement: Additional file 3 — Cis-element analysis of the APUM5 promoter. A 1.3-kb promoter region was isolated using Athena analysis tools (http://www.bioinformatics2.wsu.edu/Athena) to analyze APUM5promoter sequences. [file 1471-2229-14-75-S3.pdf]

# Additional file 4

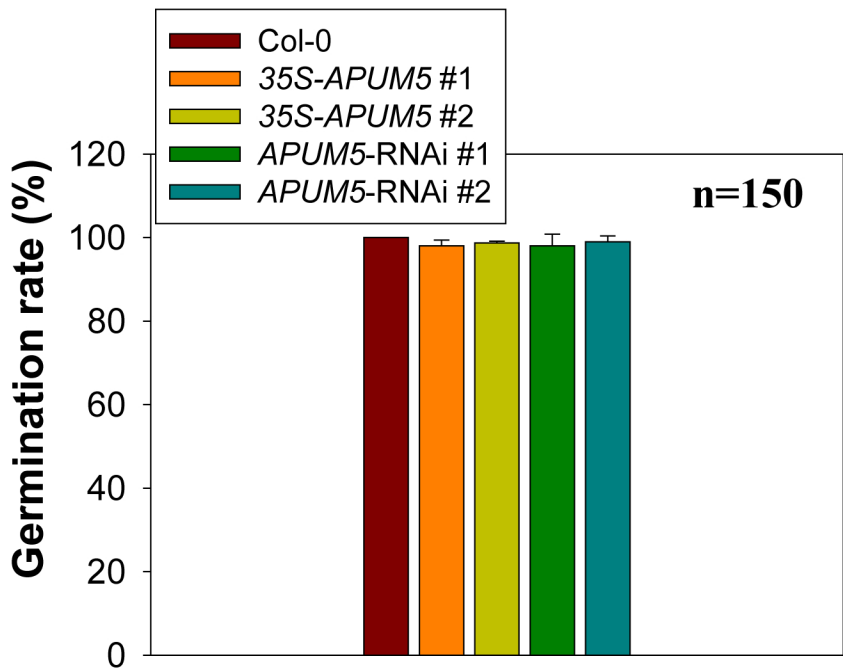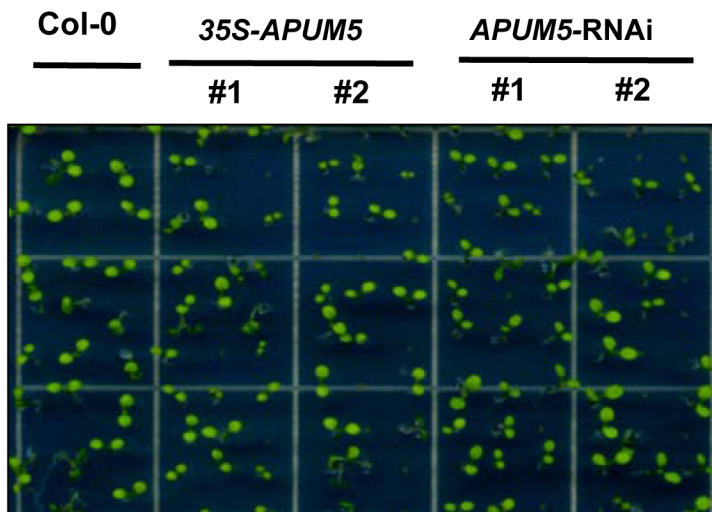

Supplement: Additional file 4 — Germination rate analysis of APUM5 transgenic plants in 1/2 MS medium plates (n = 150). [file 1471-2229-14-75-S4.pdf]

Additional file 5

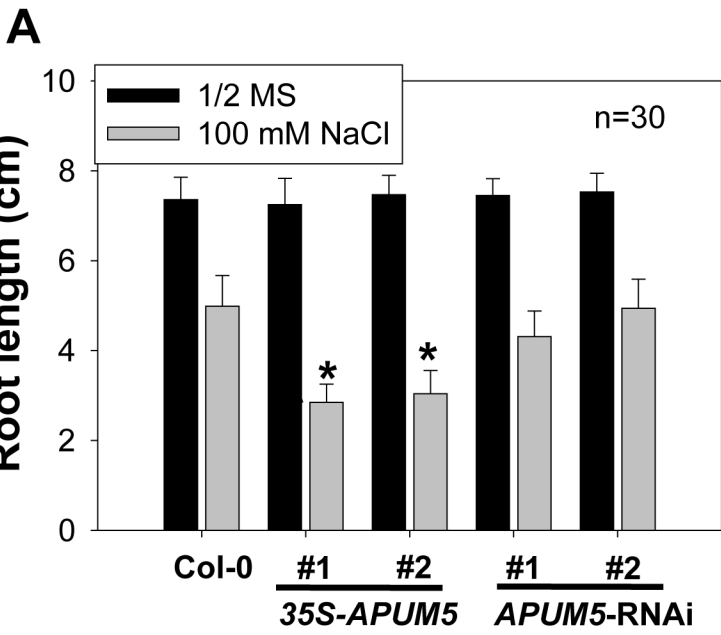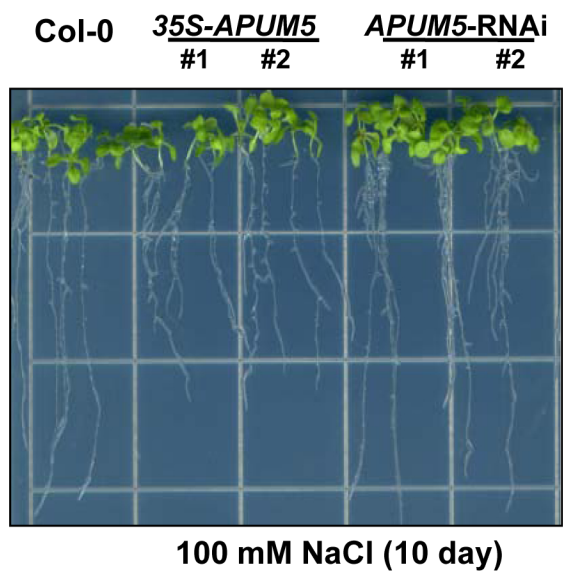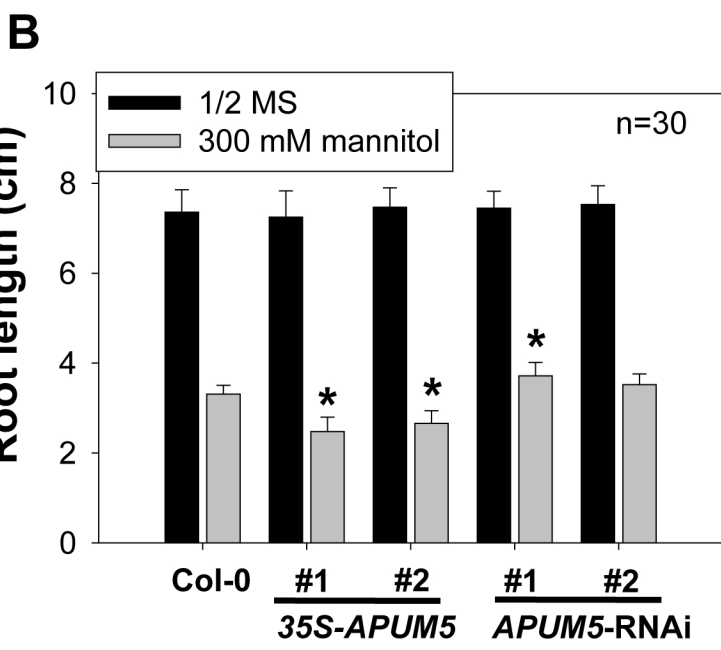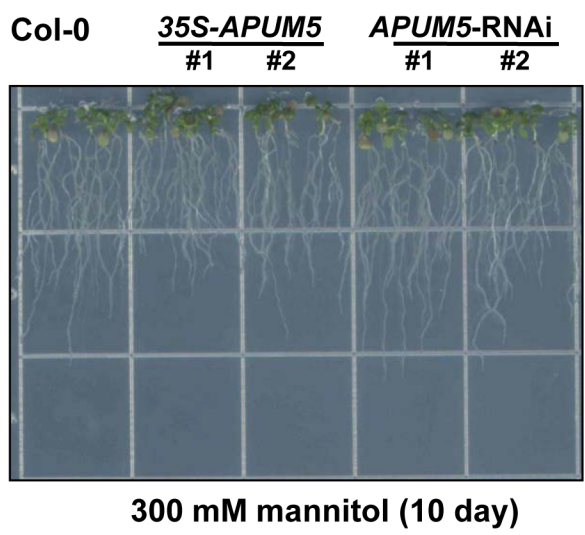

Supplement: Additional file 5 — Salt and mannitol sensitivity of wild-type and APUM5 transgenic plants. (A) The effect of 100 mM NaCl on primary root elongation. (B) The effect of 300 mM mannitol on primary root elongation. Seeds were germinated for 3 days on 1/2 MS medium, and the seedlings were transferred (n = 30, triplicates) to 1/2 MS containing 100 mM NaCl or 300 mM mannitol. Primary root length was measured at 10 days after transfer. [file 1471-2229-14-75-S5.pdf]

Additional file 6

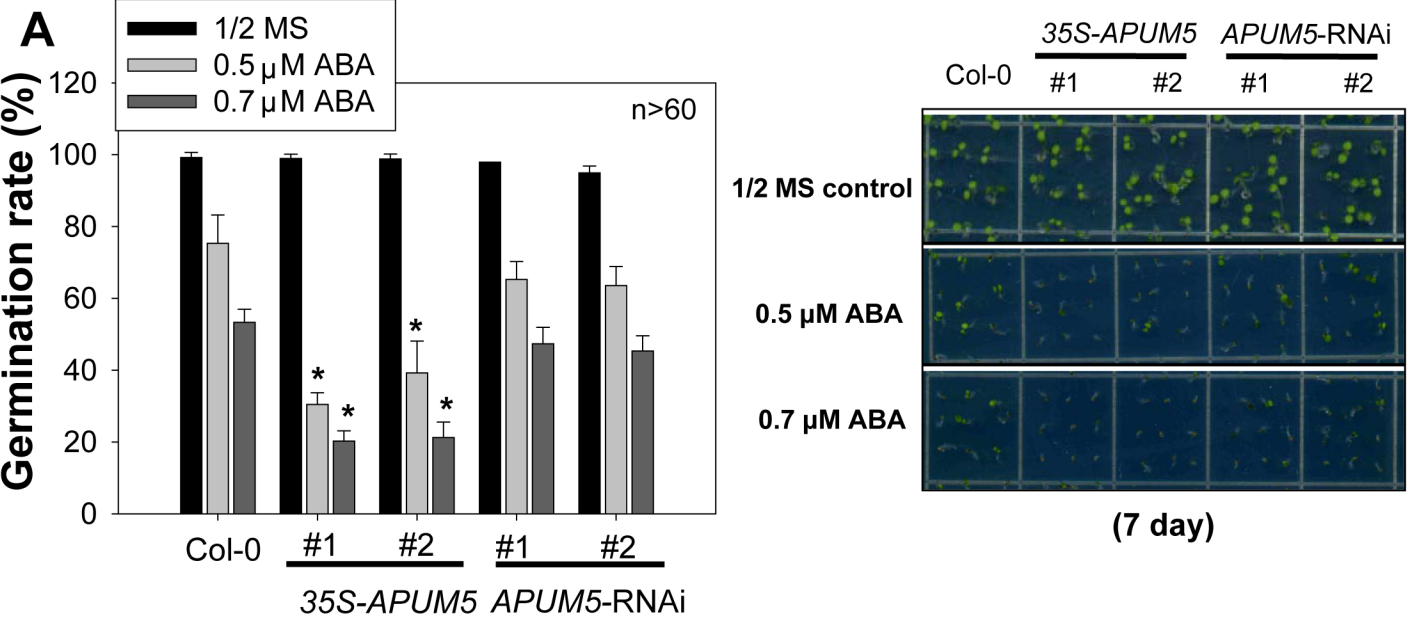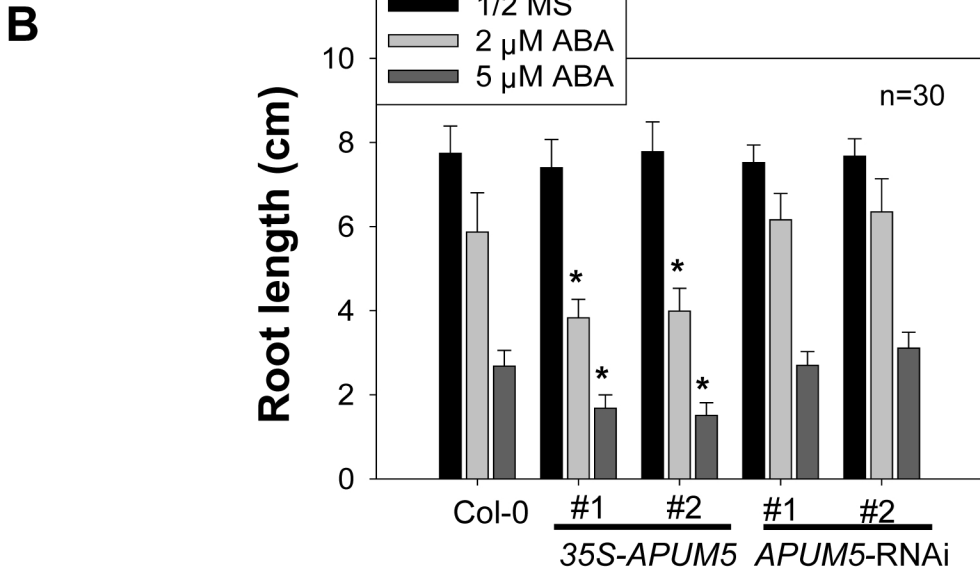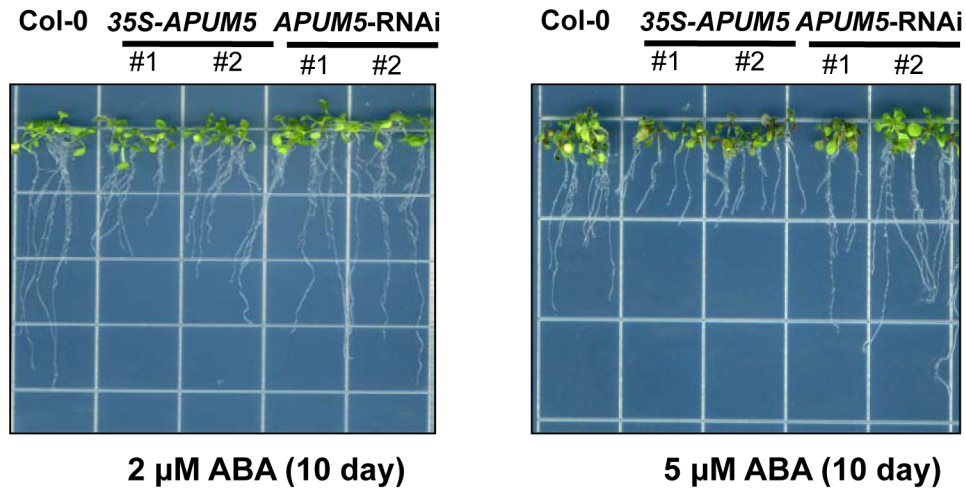

Supplement: Additional file 6 — ABA effect on seed germination and primary root growth in wild-type and APUM5 transgenic plants. (A) The effect of ABA on seed germination. Seeds were germinated and grown on 1/2 MS medium containing 0.5 and 0.7 μM ABA for 7 days, and seedlings with green cotyledons were counted (n > 60, triplicates). (B) The effect of ABA on primary root elongation. Seeds were germinated for 3 days on 1/2 MS medium, and the seedlings were transferred (n = 30, triplicates) to 1/2 MS medium containing 2 and 5 μM ABA, respectively. Primary root length was measured at 10 days after transfer. [file 1471-2229-14-75-S6.pdf]

Additional file 8

A

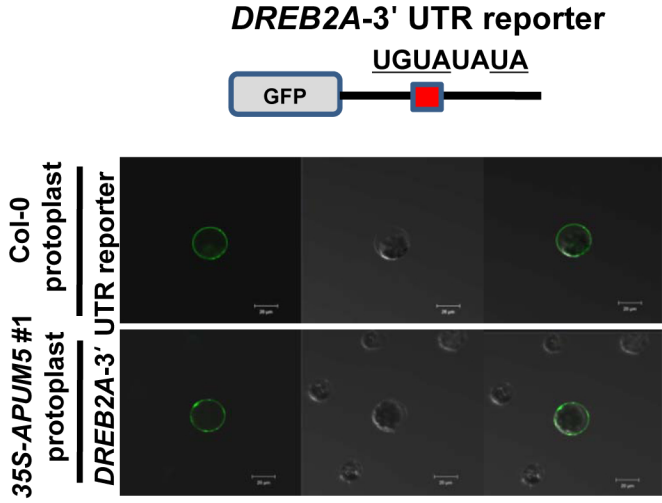

B

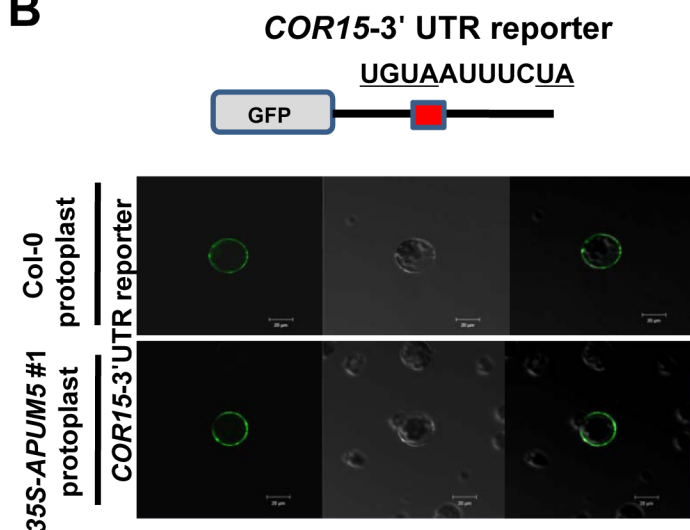

C

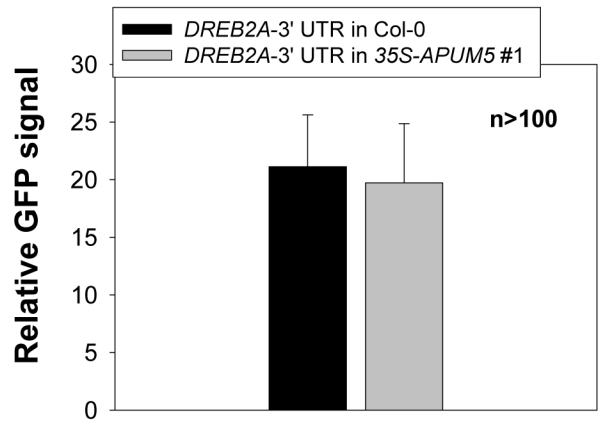

D

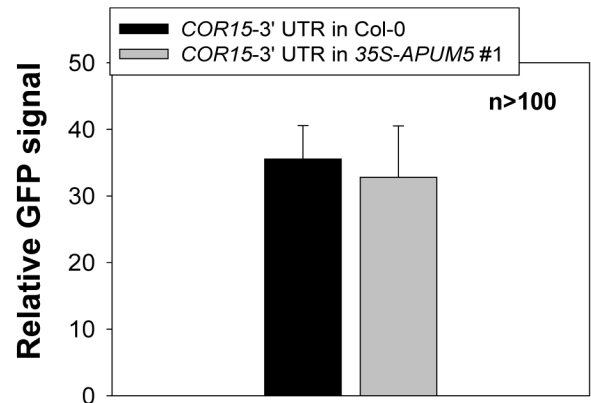

E

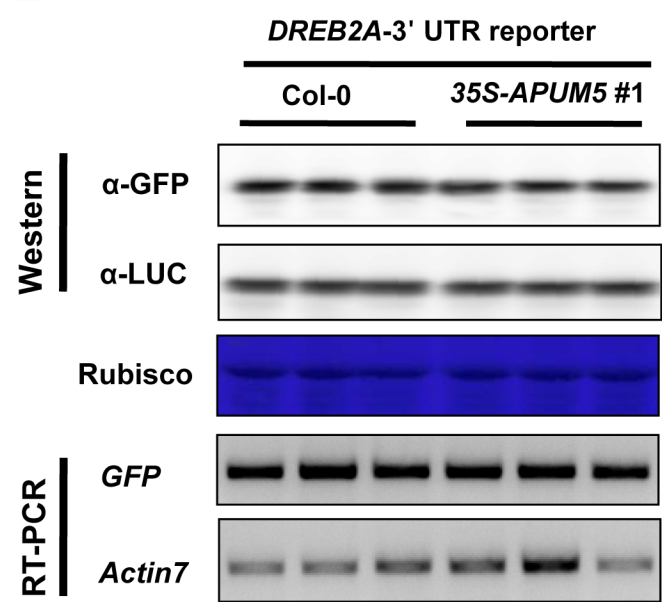

F

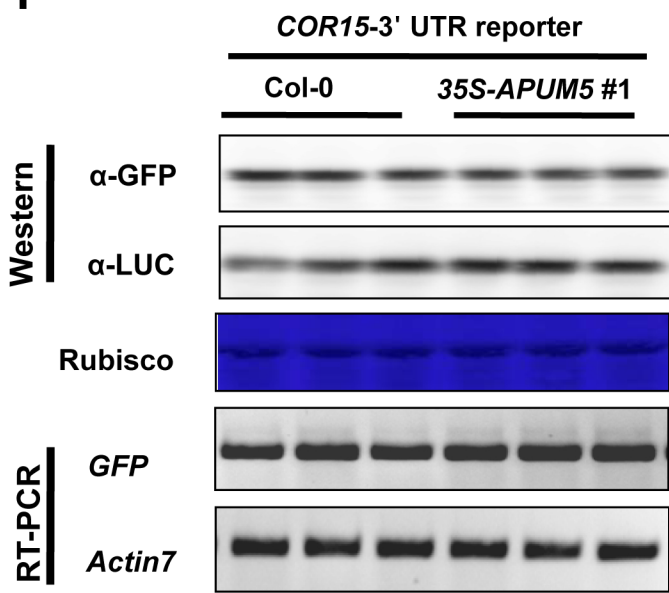

Supplement: Additional file 8 — Reporter assay. (A, B) Protoplasts of Col-0 and 35S-APUM5 transgenic plants were transformed with the DREB2A-3′ UTR reporter or the COR15-3′ UTR construct by PEG-mediated transformation. GFP signals were detected by LSM 700 confocal microscopy under identical conditions. (C, D) GFP signal intensity of the DREB2A-3′ UTR and COR15-3′ UTR reporters was quantified with Zen software of the LSM 700 confocal microscope and ImageJ software (rsbweb.nih.gov/ij/). Error bars represent ± SD. (E, F) Western blot and RT-PCR analyses were performed with protoplasts of Col-0 and 35S-APUM5 transgenic plants transformed with the DREB2A-3′ UTR reporter or the COR15-3′ UTR construct. Protein and RNA samples were extracted from four-independent experiments. Rubisco was used as a protein loading control and AtActin7 was used as the internal control. [file 1471-2229-14-75-S8.pdf]
