# Supplementary material for: APUM5, encoding a Pumilio RNA binding protein, negatively regulates abiotic stress responsive gene expression
Source: BMC Plant Biol. 2014 Mar 25;14:75. doi: 10.1186/1471-2229-14-75 (PMC3986970; doi:10.1186/1471-2229-14-75)
Supplement: Additional file 7 — Expression analysis of some abiotic stress-responsive genes that do not have a putative APUM5 target site in APUM5 transgenic plants upon drought stress. (A) mRNA levels were determined by qRT-PCR analysis using total RNAs isolated from control and 6 h drought-stressed detached leaves. Error bars represent ± SD (n = 3). (B) The 3′ UTRs of abiotic stress-responsive genes were analyzed using the Arabidopsis Information Resource (ftp://ftp.arabidopsis.org/home/tair/Genes/TAIR10_genome_release/) and the 3′ UTR sequence database UTRdb (http://utrdb.ba.itb.cnr.it/). Red indicates ‘UGUA’ tetranucleotide Puf target motif. [file 1471-2229-14-75-S7.pdf]

# Additional file 7

**A**

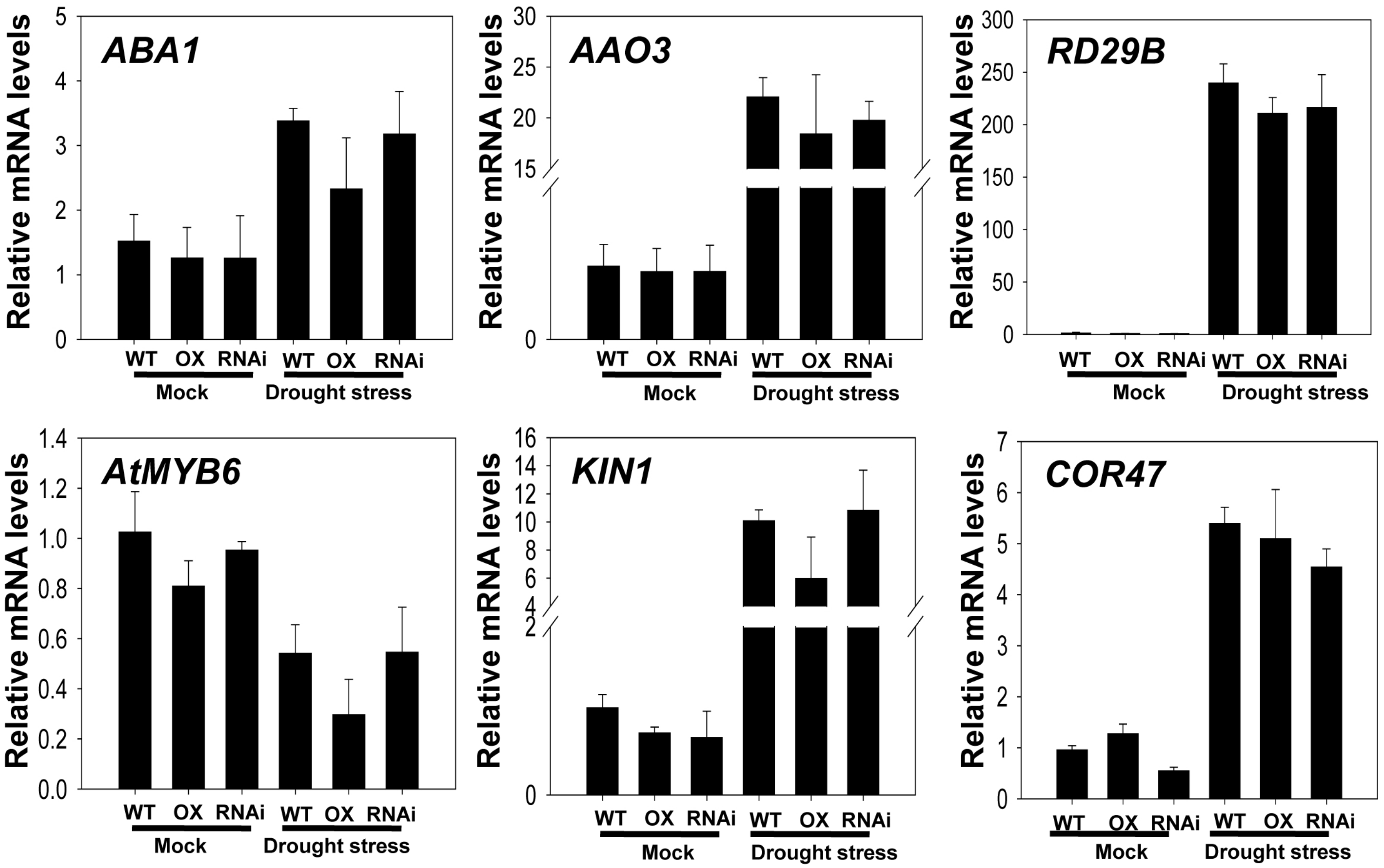


**B**

>ABA1 (AT5G67030); 5' to 3'
CGAAGGAAGAAGAUAUAGAGCAACACCGAAUUUUCCCGCGCGGUUUAGAUCGUCCGACAUCAUCGA
GUUUGGUUCAGAUAAGAAGGCGGCGUUUAGGGUGAAAGUAAUCAGGAAAACUCCGAAAUCGACGA
GGAAGAAUGAGAGUAACAACGAUAAAUUACUUCAGACAGCUUGAAAUAAGUAAACCGAUGGUGAA
AUUAAGUAAUUUUAAUCGGUUUGGCAGAUAAUUUGAGUAGUAAUUUUCUCAAAAGAAAAAAAAUC
UGCUUUCAGCUAUUCCUUGCAUGACAA**UGUAUAUAUA**GGUCUGAAACAAAAUAUAAAUUAUACAA
GACAUUAAUCUUCAUUUCUUCAACUUCCAGGCAACAAUAAUCUAAUUUUGAUUACUAAAAGGUAAU
UAUAUU

>AtMYB6 (AT4G09460); 5' to 3'
UAAUUUGUCAAAAAAAUCCCAAAAAAUGGGUUUUGUUACUUCAUUUUUUUUUUUGCUUGAGUCCU
UUUGGAUAAAUAUAGAAAUUCUUUACUUCAGUUUCUUUGGUAUUUUUGUUUUAGUUUUUGUUCAU
AUUACCUUUUGAAGAAAAAGAAAAAAAAACGAUACAUGGUUUCCAAAAUAAUUUUAUGGAAAUAUA
UAAGAAUAUUACACCAUUGUCUAGG

>KIN1 (AT5G15960); 5' to 3'
CGAUUCGGGUCAAAUUUGGGAGUUAUAAUUUCCCUUUUCUAAUUAACUGUUGGGAUUUUCAAAUA
AACGAUCUUUGAUCAAGAAUUGCAUUAUAUAUAUAUAAAUAUAUUGCAAAAUUAUUAGACGAGCC
AAACUUAUAUUCAAAUAAUGUUUUAUCUAUUUUAAAAAAUAUUCUUUAUGCGAAAGAUCAAACUC
CCCAAACGAUGUUAAAUGGAUCACGAUACAUAAGUCGAUCCGAAUUGUUGAAGUUUUCU

>AAO3 (AT2G27150); 5' to 3'
AGGCAAAGCAAGUAAGCAACUCUCGUUGCUGCUAAGUUACAGUGUUACAUAUACUAAAGUUAUUCA
GAUUUUAUAUACAGCAAAAAUAAUUGCUUUGUGCAUAACUAAUGGUUUCAAAACCAAAUCAAUAA
AUCAAAUAUUGAAUGU

>COR47 (AT1G20440); 5' to 3'
GCAAAAUGGUUAAAGAAUGAAUAAUGAUGUGGGAGUGGGACAUUCGCUGUGUUUUGUGAUCAUUA
UCUUUCUCUUUUAAGUUGUUAUUGUGGCUUUCGUUGAUUGCAUUUGAUCCUUUAUUUUGUAAUUU
CAUUCUAUCUUUUAUAUAAAGUUUGCAUAUGGUUUAUACUUAAAUGUUUUCGAGUUUGUGUAAGU
UGUGUGUGUAGGAGGAUUUCAUUUGGUUAUCCCAUAACACUCAUCGUAUACUCACUCUUACGUUUG
GUGAUUACGUUAUUCACAUGAUCACCUCGAAUUGUCAAAAAAGUUUCAAAUG

>RD29B (AT5G52300); 5' to 3'
AGAUUUGGGGUUUUGCUUUUGAAUGUUUGUGUUUUUGUAUGAUGCCUCUGUUUGUGAACUUUGAU
GUAUUUUAUCUUUGUGUGAAAAAGAGAUUGGGUUAAUAAAAUAUUUGCUUUUUUGGAUAA
