# Supplementary material for: APUM5, encoding a Pumilio RNA binding protein, negatively regulates abiotic stress responsive gene expression
Source: BMC Plant Biol. 2014 Mar 25;14:75. doi: 10.1186/1471-2229-14-75 (PMC3986970; doi:10.1186/1471-2229-14-75)
Supplement: Additional file 9 — Coupled RT and qRT-PCR primers used for expression analysis of abiotic stress-related genes. [file 1471-2229-14-75-S9.pdf]

Additional file 9

Coupled RT and qRT-PCR primers

Gene Name	Forward (5' to 3')	Reverse (5' to 3')
APUM5	GGTCAAGTCGCCACTCTTTC	CTCCAGGACATGCTGAGTGA
APUM6	CATTGTTCAGCCGAGTGAGA	TGAGTCAGCCCACTGTCTTG
RAB18	CAGCAGCAGTATGACGAGTA	CAGTTCCAAAGCCTTCAGTC
COR15	GGCCACAAAGAAAGCTTCAG	CTTGTTTGCGGCTTCTTTTC
RD22	CCGGTAAAAGAACCGACGTA	AAAGGGTTTGCTCCTGGTTT
ERD10	TCTCTGAACCAGAGTCGTTT	CTTCTTCTCACCGTCTTCAC
DREB2A	AAGGTAAAGGAGGACCAGAG	ACACAACCAGGAGTCTCAAC
ABI4	TCAATAACTCATCCACCGCCGTTG	AGGCCAAATGGTCGAAGATCCATC
ABA1	GTGATCGGATTAACGGTCTCGTTG	TGACGCCGCAGGAGTGAAAGTAT
AAO3	TCGGCGAGTACATTGTATAAGCCA	ACTTCCACCTCGCTGACTCCAACT
RD29B	CGCCACGGTCCGTTGA	TCCACCGGAATCCGAAAAC
AtMYB6	AAGTCTGACAGTGATGAGAGGAGC	AGTCTCATTTATAAGATTCACAAG
KIN1	ACCAACAAGAATGCCTTCCA	CCGCATCCGATACACTCTTT
COR47	GGAGTACAAGAACAACGTTCCCGA	TGTCGTCGCTGGTGATTCCTCT
Actin7	AATGGTGAAGGCTGGTTTTG	TGCCTCTGTGAGTAGAACTG
